# Age-related changes and selective disappearance shape variation in bold-shy continuum in guppies

**DOI:** 10.1093/beheco/arag020

**Published:** 2026-02-25

**Authors:** Magdalena Herdegen-Radwan, Jarosław Raubic

**Affiliations:** Institute of Environmental Biology, Faculty of Biology, Department of Behavioural Ecology, Adam Mickiewicz University, Uniwersytetu Poznańskiego 6, 61-614 Poznań, Poland; Institute of Environmental Biology, Faculty of Biology, Population Ecology Group, Faculty of Biology, Adam Mickiewicz University, Uniwersytetu Poznańskiego 6, 61-614 Poznań, Poland

**Keywords:** behavioral type, personality, pace-of-life syndrom, senescence, survival, additive variance, maternal effects

## Abstract

The persistence of animal personalities within populations contradicts the optimality principle, assuming a single optimal trait value for all individuals. With the advance of research on the maintenance of animal personalities, 2 types of data have emerged as particularly needed: (i) longitudinal data, allowing to distinguish population-level and cohort-level variance in personality traits, and (ii) estimates of additive genetic variance in personality traits and their genetic correlations with life history traits. While longitudinal studies are beginning to emerge, most research to date has relied on behavioral measures at a single life-stage, limiting our understanding of the interplay between ages and genotypes. To address this gap, we employed a 3-generations pedigree design in a captive guppy (*Poecilia reticulata*) population, measuring boldness, a behavioral trait showing among-individual variance, at different stages of ontogeny and collecting long-term survival data. This design enabled us to investigate the genetic contribution to variance in boldness and survival and examine how it changes across the lifespan in the behavioral trait. Our results show that boldness decreased with age. Further, we found support for phenotypic covariance between boldness and survival, resulting in a negative association between the traits. Additive genetic effects contributed to both boldness and survival, and we found negative genetic correlation between those traits, in line with POLS scenario. Lastly, average boldness was higher in males than females, while the contribution of additive genetic variance did not differ across sexes. Our findings highlight the complex, dynamic interplay of age, genotype, and sex in shaping individual behavior.

## Teaser

Being bold can have advantages in many animals, such as helping them find mates. However, some individuals are shy. Our long-term study of guppies shows that bold individuals have shorter lifespans, suggesting that being bold comes with disadvantages. We also found that guppies become less bold as they age. These factors help explain why both bold and shy personalities continue to exist in populations.

## Introduction

Over the last 30 years a large number of studies have documented the presence of behavioral or personality traits, ie behaviors that consistently differ among individuals over time and across different contexts. Such behavioral consistency of individuals results in the maintenance of different behavioral types or personalities, ie individual average behaviors (Sih et al. 2004; Réale et al. 2007), within populations, in a wide range of taxa (Bell et al. 2009; Stamps and Groothuis 2010; Carter et al. 2013). This has led to the conclusion of their universality and raised questions about the mechanisms that maintain different behavioral types within populations. As a result, phenotypic vs. genetic determinants of personalities have been investigated, revealing that additive genetic variance often substantially contributes to personality traits. A mean heritability estimate of 0.3 for personality traits has been reported across species (Réale et al. 2007; Dochtermann et al. 2015). In parallel, an increasing number of studies have shown links between personality traits, such as boldness or aggression, and fitness (reviewed in, eg Smith and Blumstein 2008), giving rise to evolutionary hypotheses for the maintenance of personalities. While such links might be expected to directionally erode variation in personality traits, different combinations of life-history and personality traits might be favored by selection within populations, representing alternative strategies to resolve evolutionary tradeoffs. This is proposed by the pace-of-life-syndrome (POLS) hypothesis (Stamps 2007; Réale et al. 2010), which posits that correlational selection or pleiotropic genetic effects drive the formation of suits of traits, that combine specific levels of life-history, metabolic and personality traits. This should result in the persistence of alternative life strategies known as fast vs. slow pace of life. Fast living organisms, characterized by high metabolic rates, rapid growth, early reproduction and short lifespans, are also expected to express proactive, ie bold, aggressive, exploratory behaviors. Thus, the POLS hypothesis assumes joint evolution of behavioral and life-history traits. While numerous studies have examined the presumed links, the results have been mixed and no general pattern has emerged. Two meta-analyses (Niemelä and Dingemanse 2018; Royauté et al. 2018) and a review (Montiglio et al. 2018) on the topic report a weak effect, not consistent across taxa and environmental conditions. Furthermore, studies that examine both reproductive success and survival, the traded off life history traits, in relation to personality traits in the same population are rare (Réale et al. 2009; Ballew et al. 2017; Santicchia et al. 2018; Gan et al. 2024; Van de Walle et al. 2024). Importantly, most studies reporting life-history-personality correlations have focused only on phenotypic associations. However, phenotypic associations may not reliably reflect genetic correlations between traits (Crespel et al. 2024), which are central to the POLS hypothesis.

Despite significant advances in the field, long-term studies covering the whole or great part of an organism's lifespan remain surprisingly scarce. However, they are crucial for drawing general conclusions about the lifelong dynamics of behavioral types: ie the stability of average behaviors in populations across time, as well as individual consistency and the resulting rank order of individuals. The few studies which have tracked behavioral traits across time have largely failed to support their stability across life stages, especially those separated by major developmental shifts (see Cabrera et al. 2021 for a review). While female and male cichlids (*Neolamprologus pulcher*) were shown to maintain in adulthood their juvenile behavioral types in terms of both population average and individual ranking, in boldness and aggression (Schürch and Heg 2010), changes in behavioral types between young and adult stages have been observed in several other species. Changes across life stages have been reported at both the population and individual levels for aggression in red squirrels (Kelley et al. 2015), exploration of great tit (Carere et al. 2005), and personality of red junglefowl (Favati et al. 2016). The emerging pattern indicates a most pronounced change in behavioral traits around the period of sexual maturation, which is hypothesized to be linked to a systemic transition, including hormonal, physiological and morphological reorganization (Cabrera et al. 2021). The scarcity of the reported cases and the lack of a general trend in observed changes highlight the need for more empirical data to comprehensively understand the phenomenon. Another explanation, proposed for the individual inconsistency of behavioral traits over time, is the increasing influence of external factors and individual experience in shaping behaviors as life progresses (Stamps and Biro 2023). Changes of average behaviors could also be attributed to differing selection pressures acting on behavioral traits at various life stages, favoring different behavioral patterns at each stage (Wolf et al. 2007). For instance, during development, risk-prone behaviors may be favored, as they allow more for efficient resource acquisition, which is crucial for tissue growth. Furthermore, as an organism ages and its expectations regarding future reproduction diminish, its behavioral strategy may shift. All the above underexplored mechanisms could disrupt the stability of behavioral types over the long-term, leading to variation at the population and individual level.

Long-term studies can also reveal whether the contribution of additive vs. other, eg maternal or environmental effects, to variance in behavioral traits changes across the lifespan. Such pattern was indeed revealed in a study by White and Wilson (2019) on guppies (*Poecilia reticulata*). In young fish, maternal effects primarily contributed to the variance in boldness, whereas in adults, the trait showed evidence of additive genetic variance, with maternal effects becoming negligible. Conversely, a study on a wild population of great tits (Class et al. 2019) found an opposite pattern, with heritability of exploration decreasing from juvenile to adult birds. Similarly, in a wild population of blue tits, heritability of 2 other behavioral traits, handling aggression and breath rate, remained stable across life stages (Class and Brommer 2015). These few contradictory reports call for further research on how the heritability of behavioral traits changes across an organism's lifespan.

Finally, combining long-term studies on behavioral traits with the investigation of their genetic underpinnings opens novel perspectives on the study of ageing, a mechanism that could contribute to the maintenance of variation in behavioral traits. Traits associated with fitness, such as many behavioral traits, are expected to decline over time—referred to as senescence (Williams 1957), and the rate of this decline is predicted to vary among genotypes, as different genotypes differ in their mutation load and may harbor divergent genetic variants with negatively pleiotropic effects. Long-term phenotypic and genotypic data on behavioral traits have the potential to uncover genotype-by-age (G×A) interactions (Class and Brommer 2015, 2016), which are considered a fingerprint of such evolved senescence. Moreover, while ageing itself should result in population-level changes in behavioral traits, genotype-dependent age-related plasticity may explain differences in behavioral types at the cohort-level. To our knowledge, only 3 studies have reported G×A interactions in behavioral traits to date, all conducted on wild tit populations (Class and Brommer 2015, 2016; Class et al. 2019), highlighting the need for further research to assess the generality of this interaction.

Boldness, understood as the propensity to take risk, is one of the behavioral traits reported to be consistently expressed by individuals in multiple species and populations (see Smith and Blumstein 2008). Here, we investigated its long-term dynamics in captive guppies to better understand the mechanisms that maintain among- and within- individual variation in this behavioral trait within populations. In the guppy population used here, boldness was previously shown to be repeatable over short periods and positively correlated with male reproductive success (Herdegen-Radwan 2019a). To understand the potential of boldness, as measured both here and in the previous study, to covary with other traits (eg age, sex, fitness), we first assessed weather it is consistent across different contexts. Consistency is a key aspect of any personality trait, particularly one with known impact on fitness, and its magnitude can provide insight into the selective pressures that maintain such traits. The confirmed high individual consistency of boldness across situations (see Results) supports its validity as an important component of the individual phenotype. Based on this premise, the present study extends our understanding by investigating the age-related dynamics of boldness, at the individual and population level, both at short- (within few days) and long-term (across life stages). We also estimated variance components contributing to the trait at different ages. Finally, we examined the potential link between boldness and survival, by examining the correlation between the 2 traits. Under the pace-of-life scenario, and in combination with the previously reported positive correlation between male boldness and reproductive success, we expected a negative correlation between boldness and survival. This would suggest a tradeoff between fast and slow life strategies, potentially driven by physiological costs associated with higher reproductive rates or overall greater energy expenditure, which could persist in the population. By investigating genetic variance in both boldness (across life) and lifespan, we aimed to assess the potential for joint evolution of these traits, as assumed but rarely tested under the POLS scenario. Throughout these analyses, we considered the effect of sex, as well as sex-specific correlations among traits, which may arise from differing selection pressures on males and females.

## Methods

### Husbandry and breeding design

The guppies used in this study originated from a captive population maintained at the Faculty of Biology at Adam Mickiewicz University (Poznań, Poland). This population descended from wild fish captured in 2002 from the Tacarigua River in Trinidad. Experimental fish were maintained under stable conditions: a temperature of 25 ± 1 °C, a 12:12-hour light/dark cycle, and were fed twice daily with live *Artemia* larvae and dry flakes. Ethical rules were followed throughout the experiment, and the study was approved by the Local Ethics Committee in Poznań (decision numbers 69/2018, 72/2022).

A paternal half-sib breeding design was employed to create a 3-generation pedigree, resulting in behavioral data from 399 individuals spanning the F1 and F2 generations. First, 50 males from the parental generation were mated with 2 mature virgin females each. The trios remained together for 6 days, after which the males were removed. Females were then transferred to individual tanks with breeding chambers, where they remained until parturition. Each female contributed a single brood and F1 offspring were housed in family groups with a maximum density of 5 fish per tank until their sex could be determined. Afterward, they were separated into same-sex family groups until their first behavioral trial. Following the trial, each fish was moved to an individual 1.5 L tank where it remained until behavioral data collection was completed. The home tanks were integrated into a ZebTec rack system (Tecniplast), which ensures identical environmental conditions. This protocol was repeated to establish and collect data from the F2 generation, for which 16 F1 males were mated to 32 F1 females, with all 3 fish in a trio coming from different families. More details on the breeding design, including basic statistics, are reported in Material S1.

Each fish was photographed on its left side under anesthesia (MS-222) to assess body size. (This was done at time point 1 for generation F1 and at time point 2 for F2, due to logistic constraints, and was accounted for by the factor “generation”; see *Statistical methods*). Body size, defined as body area excluding fins, was measured using Image J software (https://imagej.net/ij/). Measuring body area as an estimate of body size is a commonly used method in guppy studies (eg Pilastro et al. 2002; Devigili et al. 2015). Body size has been reported in various species to covariate with behavioral traits (Mayer et al. 2016; Ferderer et al. 2022), thus accounting for body size was expected to modify the amount of variance in boldness.

### Behavioral tests and data collection

The level of boldness was assessed using an emergence test previously shown to be repeatable in this population (Herdegen-Radwan 2019b). Briefly, the fish was placed in a small, dark refuge chamber within an unfamiliar aquarium, via a hole in the ceiling. After a 3-min acclimation period, the door of the chamber was opened, allowing the fish to swim out into the open, illuminated space. The time taken to emerge was recorded, with a maximum score of 300 s assigned to fish that did not emerge within 5 min of the door opening. Fish that emerged sooner were considered bolder. In both generations (F1 and F2), fish underwent the following sequence of emergence tests. (i) Time point 1 (4 to 5 months): Each fish underwent 2 emergence tests spaced 2 to 3 days apart. (ii) Time point 2 (7 to 8 months): Each fish was subject to 3 emergence testes over 3 consecutive days: (a) a standard emergence test described above, (b) an emergence test with a small container with a conspecific female (visual contact only) placed opposite to the chamber entrance, and (c) an emergence test with a novel object (a blue plastic Scotch tape dispenser) placed opposite the chamber entrance. The order of these test variants was randomized across individuals. (iii) Time point 3 (11 to 12 months): Fish were subject to the same 3 variants of emergence test as at time point 2, in a newly randomized order. (iv) Time point 4 (15 to 16 months): Surviving fish underwent 2 standard emergence tests identical to those at time point 1. Throughout the manuscript, time points 1 to 4 refer to the corresponding trial sessions and fish ages. The boldness test conducted in an empty aquarium is referred to as the standard test. Boldness scores from time points 2 and 3 were used to estimate individual consistency across contexts to investigate whether boldness is consistent across different life aspects of guppies. The relationship between boldness and survival was analyzed based on boldness data from time point 1. Boldness scores from all 4 time points were used for all other analyses, including the estimation of repeatability, the analysis of variance components (additive genetic and maternal identity effects), the assessment of genotype-by-sex (G × S) and genotype-by-maternal identity (G × M) interactions, and the examination of age effects on boldness, including genotype-by-age (G × A) interactions. Age was categorized into 4 classes corresponding to the time points of behavioral tests.

Survival was defined as surviving or not surviving (a binary variable) until the final boldness test, which was performed at a relatively old age (15 to 16 months). By this age, 67% of the fish survived (Table 1), likely nearing or past the threshold of their reproductive phase (Reznick et al. 2006). According to life history theory, selection on fitness-related traits is expected to cease at the post-reproductive stage. Therefore, the lifespan proxy used here aligns with the study's goal of identifying any contributions of boldness to potential reproduction-survival tradeoffs.

**Table 1:** The table shows the number of individuals scored for boldness at each time point (age), separated by sex.

Time point	Females	Males	Sum
*1*	210	189	399
*2*	200	176	376
*3*	180	140	320
*4*	156	110	266

For each age class, the number of females, the number of males, and the total number of individuals (Sum) are reported. All measurements were included in Model 1; only female or male measurements were used in the sex-specific Models 2 and 3; and only scores from the first time point were included in Model 4.

### Statistical methods

All statistical analyses were performed in R v. 4.4.1 (R Development Core Team 2008). Models were constructed using the Markov Chain Monte Carlo (MCMC) algorithm, with Bayesian inference employed to estimate posterior distributions, as implemented in the MCMCglmm R package (Hadfield 2010). Results were visualized using the ggplot2 package (Wickham 2016). All continuous traits were mean-centered and scaled to standard deviation units. For fixed effects, the posterior distribution means with 95% highest posterior density intervals (95% HPDI) were reported. While posterior distributions nonoverlapping zero are interpreted as significant, we also provided *pMCMC* values—a Bayesian measure of significance, representing the probability that the posterior distribution is not different from zero. Variance components, being inherently positive, cannot be strictly tested for significance in the same way. Thus, we assessed the biological importance of the variance components by examining if their posterior distribution is clearly distinct from zero. To this aim, as a measure of central tendency for posterior distributions of variance components, we reported not only the posterior median with 95% HPDI, as recommended by Pick et al. (2023), but also the mode. Weakly to moderately weakly informative priors were used in the models (V = 1, nu ranging from 0.002 to 5). Priors for residual and random effect covariances were set as identity matrices, assuming uncorrelated effects with unit variances. Model robustness was confirmed by rerunning analyses with slightly modified priors. Adequate mixing of the MCMC chains was verified by inspecting trace plots of variance components. Analyses were run long enough to achieve effective sample sizes of several thousand for each model term, with most variance components and fixed effects typically exceeding 10,000, indicating reliable posterior estimates (Wilson et al. 2010; de Villemereuil 2018). Each model was run for 1 million iterations (nitt), which was increased up to 150 million if the above conditions were not met. We discarded the first 1,000 to 10,000 steps (burnin), and results were stored every 5 to 100 steps (thin).

We first estimated cross-context correlations (Model 1 in Table 2, where model formulae for all models are reported) to determine if and how individuals change their behavior across different contexts (see Material S2  *Methods* for details of model specification and estimation of among-individual correlations). Inter-individual correlations of boldness scores across contexts were high, indicating that the test captured a consistent behavioral trait in all 3 scenarios. Thus, we used data from all trials in subsequent analyses and treated them as a single measure of boldness to increase the precision of our estimates.

**Table 2:** Model formulae.

Model	Dataset	Model formula
1	TP 1–4; F + M	$y_{ijk}^{C}=\;\beta_{0}^{C}+\;\beta_{\mathrm{Sex}}^{C}\mathrm{Se}x_{k}+\;\beta_{\mathrm{Age}}^{C}\mathrm{Ag}e_{ij}+\;\beta_{\mathrm{Gen}}^{C}\mathrm{Ge}n_{k}+\;u_{ID(k)}^{C}+\;e_{ijk}^{C}$ ; *C ∈ {S, F, O}*
2a	TP 1–4; F or M	$y_{ijk}=\;\beta_{0}+\;\beta_{\mathrm{Age}}\mathrm{Ag}e_{ij}+\;\beta_{\mathrm{Gen}}\mathrm{Ge}n_{k}+\;\beta_{\mathrm{Trial}}\mathrm{Tria}l_{ij}+\;\beta_{\mathrm{Con}}\mathrm{Co}n_{ij}+\;u_{ID(k)}^{\left(j\right)}+\;e_{ijk}^{\left(j\right)}$
2b	TP 1–4; F or M	$y_{ijk}=\;\beta_{0}+\;\beta_{\mathrm{Age}}\mathrm{Ag}e_{ij}+\;\beta_{\mathrm{Gen}}\mathrm{Ge}n_{k}+\;\beta_{\mathrm{Trial}}\mathrm{Tria}l_{ij}+\;\beta_{\mathrm{Con}}\mathrm{Co}n_{ij}+\;u_{ID(k)}+\;u_{ID(k)}^{\left(j\right)}+\;e_{ijk}^{\left(j\right)}$
3a	TP 1–4; F or M	$y_{ijk}=\;\beta_{0}+\;\beta_{\mathrm{Age}}\mathrm{Ag}e_{ij}+\;\beta_{\mathrm{Gen}}\mathrm{Ge}n_{k}+\;\beta_{\mathrm{Trial}}\mathrm{Tria}l_{ij}+\;\beta_{\mathrm{Con}}\mathrm{Co}n_{ij}+\;u_{ID(k)}^{\left(j\right)}+\;a_{\mathrm{animal}(k)}^{\left(j\right)}+\;m_{\mathrm{mother}(k)}^{\left(j\right)}+\;e_{ijk}^{\left(j\right)}$
3b	TP 1–4; F or M	$y_{ijk}=\;\beta_{0}+\;\beta_{\mathrm{Age}}\mathrm{Ag}e_{ij}+\;\beta_{\mathrm{Gen}}\mathrm{Ge}n_{k}+\;\beta_{\mathrm{Trial}}\mathrm{Tria}l_{ij}+\;\beta_{\mathrm{Con}}\mathrm{Co}n_{ij}+\;\beta_{\mathrm{Size}}\mathrm{Siz}e_{k}+\;u_{ID(k)}+\;e_{ijk}$
3c	TP 1–4; F or M	$y_{ijk}=\;\beta_{0}+\;\beta_{\mathrm{Age}M}\mathrm{Age}M_{ij}+\;\beta_{\mathrm{AgeWH}}\mathrm{AgeW}H_{ij}+\;\beta_{\mathrm{Gen}}\mathrm{Ge}n_{k}+\;\beta_{\mathrm{Trial}}\mathrm{Tria}l_{ij}+\;\beta_{\mathrm{Con}}\mathrm{Co}n_{ij}+\;u_{ID(k)}+\;e_{ijk}$
4	TP 1; F or M (no $\beta_{\mathrm{Sex}}^{T}\mathrm{Se}x_{k}$); F + M	$y_{ijk}^{T}=\beta_{0}^{T}+\;\beta_{\mathrm{Sex}}^{T}\mathrm{Se}x_{k}+\;\beta_{\mathrm{Gen}}^{T}\mathrm{Ge}n_{k}+\;a_{\mathrm{animal}(k)}^{T}+\;e_{ijk}^{T}$ *; T* ∈ {Survival, Boldness}

In multivariate Model 1, C denotes context, with levels “S”, “F”, and “O”, corresponding to emergence test setups: standard, with female, and with object, respectively. In Model 4, boldness is averaged across 2 trials at time point 1 and modeled using a Gaussian distribution, and the response variable is bivariate, comprising boldness and survival. In Model 3c, *AgeM* and *AgeWH* represent the between-individual and within-individual effects of age, respectively. For fixed effects, *Gen* and *Con* refer to generation and context, respectively. For random effects, ID refers to individual identity (accounting for repeated measures), *animal* encodes additive genetic effects, and *mother* represents maternal identity effects. β denotes fixed-effect regression coefficients, and u denotes random effects. Indices *i*, *j*, *k* denote observation, time point and individual, respectively. The *Dataset* column indicates the observations included in each model (TP, time point; F, females; M, males). In Models 1 to 3, the response variable *y* refers to boldness, which consists of repeated-measures scores and is modeled as a censored Gaussian variable.

Next, repeatability of boldness was estimated over short- (few days, Models 2a, 2b in Table 2) and long-term (across life stages, Model 2b in Table 2), which allowed us to assess if boldness constitutes a consistent behavioral trait across lifespan. Boldness repeatability was calculated separately for males and females, given that sexes often differ in behavioral consistency (meta-analysis of Bell et al. 2009). Details of model specification and repeatability estimation are provided in Material S3 (*Methods*).

For estimating the contributions of the variance components: additive genetic (V_A_) and maternal identity (V_M_), to boldness, we adopted the animal model approach (Models 3a, Table 2). All boldness scores at all 4 time points were used as response. Boldness scores were right-censored at 300 s, corresponding to the maximum trial duration. Individuals that did not emerge from the shelter within the observation period were assigned a censoring time of 300 s and coded as right-censored. These data were analysed using a censored Gaussian distribution (family = “cengaussian”), which allows appropriate estimation of effects in the presence of censoring. The random term “identity” (ID) was included to account for repeated measures. Another random effect, “animal”, was used to account for additive genetic variance; this effect represents the same individuals as “identity”, but is linked to pedigree information, incorporating both mother and father identities. Maternal effects were accounted for by fitting a separate random effect, “mother”, representing maternal identity. Given our experimental design, in which each female contributed a single brood of full siblings housed in family groups, the estimated maternal effects likely represent a combination of maternal and early environmental influences. Because we cannot fully disentangle these sources of variance and expect maternal identity to contribute more strongly than the standardized rearing environment, we refer to this component as V_M_ throughout the manuscript, acknowledging that it may include some confounded early environmental variance. Random effects (ID, animal and mother) were modeled as age-specific, with unstructured variance–covariance matrices across ages, while residual covariances across time points were constrained to zero. Separate models were built for each sex to test whether V_A_ and V_M_ contribute differently to the expression of boldness in males and females and across the lifespan. Age- and sex-specific heritability (*h*^2^) and contribution of maternal identity effects (*m*^2^) to boldness were estimated as the proportion of V_A_ or V_M_ at a given time point relative to the total phenotypic variance (V_P_) at that time point. V_P_ was defined as the sum of all estimated variance components—additive genetic, maternal, among-individual, and residual variance—conditional on the fixed effects. Fixed effects included age, generation, trial (the sequential number of repeated boldness tests within a time point) and context. To identify life stages at which boldness significantly changed, contrasts were calculated for all pairs of age classes. A modified version of the above models (Models 3b in Table 2) was run to test the effect of body size on boldness. This model included body size as a fixed effect and used a simplified random structure, including only “identity”. This model was fitted separately to avoid confounding the estimates of boldness heritability by including the size measure (Wilson 2008).

Results from Models 3a were also used to estimate genotype-by-sex (G×S) interactions in boldness, by calculating the difference in V_A_ between males and females, separately for all age classes. A posterior distribution of the difference distinct from zero would support the presence of a G×S. The same approach was taken to estimate maternal effects-by-sex (G×M) interactions at the 4 time points. Finally, to test whether boldness is undergoing senescence, we estimated differences in additive genetic variances across age classes, and calculated genetic correlations across ages. Evidence for G×A would be indicated by significant differences in V_A_ among age classes, and/or genetic correlations significantly lower than 1.

To assess the potential of boldness and survival to evolve jointly, as predicted under the POLS scenario, we estimated the correlation between the traits by fitting a bivariate animal model (Model 4, Table 2). Survival till the final behavioral test was modeled as a binary response (survived/not survived). The average boldness at time point 1 and survival were modeled as a bivariate response. While we acknowledge that using averaged, instead of raw, boldness scores may lead to overestimation of the strength of correlations, we were mostly interested to reveal whether those correlations exist, which is a key assumption of the POLS hypothesis. Generation was included as fixed effect. Both the random effect “animal” and the residuals were modeled using unstructured variance–covariance matrices across response traits. As maternal effects on boldness were negligible for both males and females at time point 1 analyzed here, they were not included in the model. Since previous research in this population reported a positive correlation between boldness and reproductive success only in males (Herdegen-Radwan 2019a), we considered the possibility that males alone are involved in pace-of-life tradeoffs or that these tradeoffs differ among sexes, as predicted (Hämäläinen et al. 2018) and observed in other species (Moschilla et al. 2019). Thus, we ran the model separately for males and females. From those models, we first calculated heritability of survival, to evaluate whether the trait could show correlated responses with boldness through genetic effects. We then estimated correlations between survival and boldness at the phenotypic and additive genetic levels. Phenotypic regression of boldness on survival was also calculated from the model by dividing the summed components of the boldness-survival covariance by the total variance in boldness. Since the phenotypic and additive genetic correlations between boldness and survival were comparable in magnitude for males and females, the final model combined both sexes, with sex included as a fixed effect.

## Results

Sample sizes across time points and sexes are reported in Table 1, providing an overview of the dataset used for model fitting. Models 1 to 3 were built using all available data points (sex-specific where indicated), whereas Model 4 was based only on data from time point 1.

Median estimates of all among-individual correlations for boldness scores across contexts (Model 1 in Table 2) were >0.94, and the lower limits of the 95% HPD intervals of their posterior distributions were all above 0.80 (Material S2: *Results*, Table S2.1, Figure S2.1), demonstrating that the ranking of individuals was maintained across contexts (full model provided in Material S2, Table S2.2). This result indicates that boldness in the studied population is a consistent behavioral trait. Given this high consistency across contexts, we combined the scores from all trials into a single boldness measure for further analyses.

**Figure 1:**
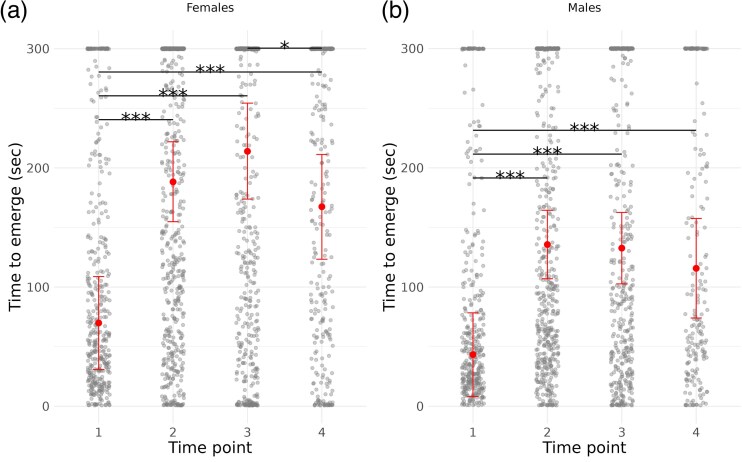
Boldness (scaled emergence time) across 4 ages (time points 1–4), plotted separately for females and males. Raw observations are shown as jittered points, and posterior medians with 95% HPD intervals from Models 3a are represented as points with error bars. Horizontal lines with asterisks above denote significant pairwise differences between time points (* *P* < 0.05; *** *P* < 0.001).

Age affected average boldness levels (Models 3a in Table 2; model results in Material S4, Table S4.1), and pairwise contrasts among age classes (Material S4, Table S4.2) revealed that the change occurred mostly between time points 1 and 2, when boldness decreased across individuals, in both sexes (all *pMCMC* < 0.001 after applying Holm-Bonferroni correction for multiple tests; Fig. 1). Moreover, there was a trend toward a decrease in boldness between ages 3 and 4 in both sexes, although the change was statistically significant only in females (Fig. 1). Trial was a significant factor influencing boldness in both sexes, with boldness decreasing from trial 1 to trial 2 (*pMCMC* < 0.001; Material S4, Figure S4.1). Average boldness did not differ across generations (*pMCMC* n.s.). Context was significant for males (*pMCMC* = 0.04), who acted less boldly in the standard than in the other 2 scenarios. Body size (Model 3b in Table 2) had no effect on female boldness, but smaller males were bolder (*pMCMC* = 0.011; full model in Material S4, Table S4.3).

Short-term repeatability of boldness (Models 2a, 2b in Table 2) was moderate but significant at all life stages in both sexes: median range for males was 0.23–0.42, and for females 0.16–0.24. Short-term repeatability across ages (Models 2b) was lower than repeatability estimated at single time points (median = 0.14 and 0.15, for females and males, respectively), suggesting reduced consistency in individual boldness between life stages. In line with this, long-term repeatability of boldness (median for females 0.03; for males 0.06) was considerably lower than both age-specific and cross-age short-term repeatability, indicating that individuals exhibit very limited consistency in their boldness levels across the lifespan. Full models are reported in Material S3 (Tables S3.2 and S3.3 for results of Models 2a and 2b, respectively), whereas all repeatability estimates with HPD intervals are provided in Table S3.1 of the same supplement.

**Figure 2:**
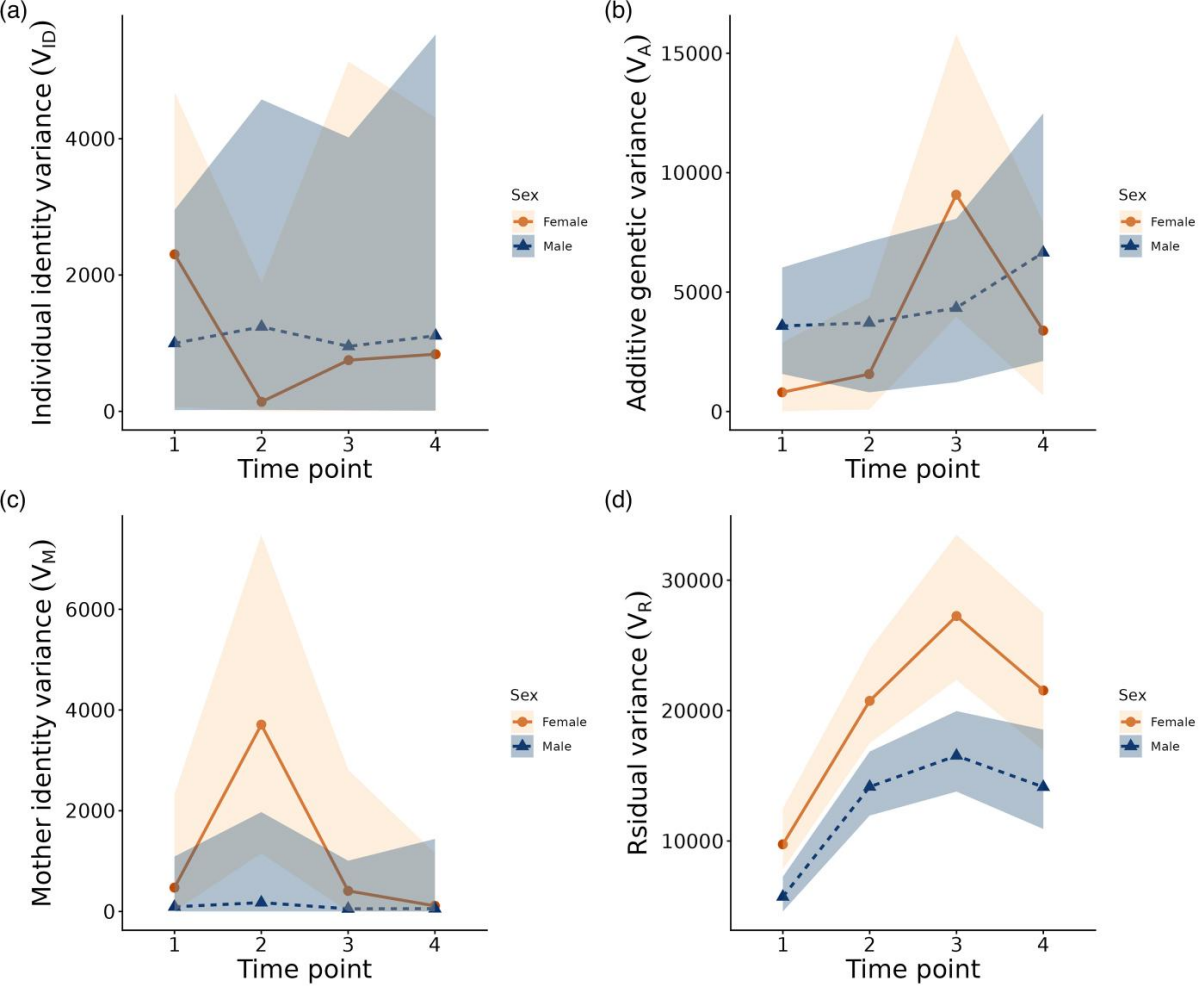
Changes in variance components across time points. Contributions of different variance components to boldness across ages, shown separately for males and females: a) individual identity (V_ID_); b) additive genetic effects (V_A_); c) mother identity (V_M_); and d) residual variance (V_R_). Lines (solid for females, dashed for males) connect posterior median age-specific values (circles for females, triangles for males), while shaded areas represent 95% HPD intervals.

Boldness heritability showed different patterns in males and females (Models 3a in Table 2). In males, heritability was generally higher and relatively stable across time points (median range: *h*^2^ = 0.19–0.34; Table 3), whereas in females it was very low at early ages and increased primarily at time point 3, reflecting an increase in additive genetic variance V_A_ (Material S4, Table S4.4; median range: *h*^2^ = 0.06–0.24, Table 3). The differences in heritability were primarily driven by consistently higher residual variance in females across life stages, combined with trajectories of change in V_A_ that, although not significantly different, showed distinct patterns between the sexes (Fig. 2, Material S4, Table S4.5). Maternal identity variance was negligible in males across all life stages and similarly low for females, except at time point 2, where it contributed moderately to female boldness (median *m*^2^ = 0.14, Table 4; Material S4, Table S4.5). Estimates of *h*^2^ and *m*^2^ of boldness at each time point, accompanied by HPD intervals, are reported in Table 3.

**Table 3:** Estimates of heritability (*h*^2^) and maternal identity effects (*m*^2^) on boldness (Models 3a) for females and males across time points 1–4 (as indicated by numbers in the *Dataset* column) are presented.

Dataset	*h* ^2^	*m* ^2^
*Females, 1*	0.059 [0.000–0.173]; 0.001	0.035 [0.000–0.139]; 0.002
*Females, 2*	0.059 [0.000–0.149]; 0.042	0.140 [0.042–0.250]; 0.134
*Females, 3*	0.238 [0.110–0.368]; 0.238	0.011 [0.000–0.057]; 0.001
*Females, 4*	0.128 [0.017–0.258]; 0.118	0.004 [0.000–0.032]; 0.001
*Males, 1*	0.337 [0.162–0.504]; 0.346	0.009 [0.000–0.079]; 0.001
*Males, 2*	0.188 [0.043–0.326]; 0.193	0.009 [0.000–0.077]; 0.001
*Males, 3*	0.196 [0.060–0.326]; 0.196	0.002 [0.000–0.032]; 0.000
*Males, 4*	0.297 [0.111–0.473]; 0.303	0.002 [0.000–0.043]; 0.000

The table reports posterior medians (±95% HPD interval) and modes.

**Table 4:** Testing for genotype-by-age (G×A) interactions using a character state approach: cross-age genetic correlations in boldness were estimated from Models 3a for females and males.

Time points	Females	Males
*1–2*	0.997 [0.155–1.000]; 0.997	0.999 [0.989–1.000]; 1.000
*1–3*	0.998 [0.549–1.000]; 0.997	0.999 [0.990–1.000]; 1.000
*1–4*	0.997 [0.455–1.000]; 0.997	0.999 [0.993–1.000]; 1.000
*2–3*	0.999 [0.960–1.000]; 1.000	0.999 [0.989–1.000]; 1.000
*2–4*	0.999 [0.953–1.000]; 1.000	0.999 [0.992–1.000]; 1.000
*3–4*	1.000 [0.990–1.000]; 1.000	0.999 [0.993–1.000]; 1.000

The table reports posterior medians (±95% HPD interval) and modes.

There was no support for a genotype-by-age (G×A) interaction in their effect on boldness. Although in females the amount of additive genetic variance increased between time point 2 and 3 (Material S4, Table S4.4), medians of all cross-age genetic correlations in boldness were all close to 1 for both sexes, indicating that the relative ranking of genotypes remained stable across life stages (Table 4).

**Figure 3:**
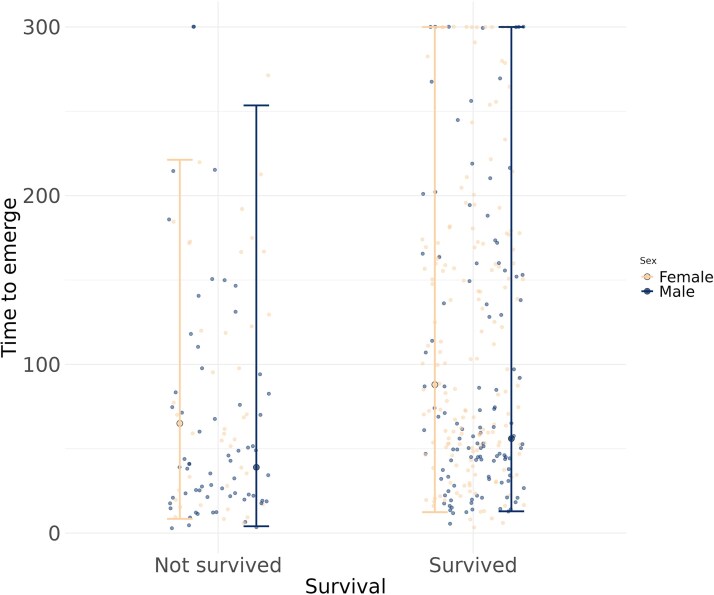
Survival (binary) until time point 4 in relation to average boldness at a young age (time point 1), measured as scaled emergence time, plotted separately for females and males. Raw observations are shown as jittered points, and posterior medians with 95% HPD intervals from Model 4 are shown in sex-specific colors (see figure legend).

Model 4 indicated that survival was significantly heritable, in both males (median *h*^2^ = 0.39 [0.04–0.80]; mode: 0.29) and females (median *h*^2^ = 0.30 [0.16–0.48]; mode: 0.28). We found indication of phenotypic (median: 0.19 [0.00–0.38], mode: 0.18) correlation between survival and boldness; the median regression estimate of boldness on survival was 0.34 [−0.04–0.74] (full model in Material S5, Table S5.1). While the 95% HPD interval for the posterior distribution of the correlation (with a lower limit of 0.002) approached zero—and that for the regression minimally overlapped it—both estimates of central tendency were clearly distinct from zero, supporting the presence of an effect. There was also support for genetic correlation between boldness and survival (median: 0.40 [0.05–0.69, mode: 0.44). These results suggest potential joint evolution of the traits through a tradeoff, as bolder individuals and genotypes had shorter survival. As a follow-up to the observed personality-related mortality, we built an age decomposition model (Model 3c; Table 2), separately for each sex, to determine whether the population-level decrease in boldness (see Model 3a) was solely due to selective disappearance of bolder individuals or also influenced by within-individual plasticity in boldness. Individual plasticity was expected given that the greatest change occurred between time points 1 and 2, ie when most individuals were alive. Indeed, boldness decline in both sexes was significantly explained by cross-age plasticity, but males also experienced an additional decrease due to selective disappearance of bolder individuals (*pMCMC* = 0.04; model description and full model results are reported in Material S5, *Age decomposition model*, Table S5.2). Finally, males survived shorter (*pMCMC* < 0.001, Model 4, Fig. 3) and were bolder than females (*pMCMC* < 0.001, Model 4, Fig. 1).

## Discussion

Using a 3-generation pedigree design, we found that boldness in our guppy population is a heritable behavioral trait. However, long-term nature of the study also allowed us to uncover the changing contribution of variance components to the trait throughout life. Boldness decreased with age, and this age-related decline was driven by a combination of phenotypic plasticity across the lifespan and the selective disappearance of bolder individuals. Indeed, consistent with the pace-of-life syndrome (POLS) hypothesis, boldness—previously shown to be positively correlated with male reproductive success in this population (Herdegen-Radwan 2019a), was negatively correlated with survival, both at the phenotypic and at the genetic level.

### Boldness repeatability

We found support for boldness consistency across contexts in the studied population, as indicated by the high cross-context correlations among individuals, which were close to 1. The contexts were designed to differ qualitatively. Although both the novel environment and novel object trials focused on novelty, they were shown not to be interchangeable in damselfish (White et al. 2013), and the third trial involved a mating/social context (for males and females, respectively). The high individual consistency across contexts suggests that boldness, as measured in this study, constitutes a stable component of guppy behavior in both sexes. This result also strengthens our conclusions about the generality of the reported effects.

Short-term repeatability of boldness was moderate at all life stages, whereas cross-age short-term repeatability was lower but similar between sexes, indicating comparable levels of temporal consistency in males and females. White et al. (2019) reported repeatability estimates comparable to ours for most risk-related behaviors measured in a captive guppy population. Long-term boldness was notably less repeatable, consistent with a trend observed across-taxa in a meta-analysis by Bell et al. (2009). This may be due to both, shifts in genetic machinery influencing the same trait at different life stages, as well as environmental effects, more likely to change over long intervals (Bell et al. 2009). This decrease in repeatability when measured over a long time scale tended to be slightly more pronounced in females, consistent with the findings of Bell et al. (2009), whose meta-analysis reported lower behavioral repeatability in females. This pattern may reflect hormonal fluctuations, as intra-individual variation in female guppy behavior has been linked to the ovarian cycle (Warren and Callaghan 1975). Overall, these results indicate substantial within-individual variation in boldness over the course of life.

Finally, at each time point—ie at the short time scale—average boldness decreased after the first trial. This effect may reflect familiarization with the test arena and a reduced motivation to emerge to explore it. Importantly, the effect disappeared over the longer time scale, ie between time points, indicating that the overall age-related pattern observed in the population is not a consequence of this trial effect.

### Additive and maternal identity variance in boldness

Boldness was moderately heritable in males and older females, its heritability falling within the range of estimates reported for personality traits (Réale et al. 2007; Dochtermann et al. 2015). These results demonstrate the presence of genetic variance in boldness, which is a fundamental assumption underlying hypotheses that explain the evolution of behavioral traits, including the pace-of-life syndrome (POLS) hypothesis. This finding supports the potential for the trait to evolve and be involved in evolutionary tradeoffs.

The heritability estimates corroborate with those reported by White and Wilson (2019), who found a non-negligible contribution of additive genetic effects to risk-related behavioral traits in adult guppies from a laboratory population. Given that White and Wilson (2019) estimated heritability jointly for both sexes, their estimates are of the same order of magnitude as the sex-averaged boldness heritability from the present study, where at young age heritability was negligible in females, but present in males. While they reported no additive genetic variance contributing to risk-taking behavior in young fish, this was measured at 1–2 months of age, whereas our tests were conducted starting at the 4th–5th month of life. Together, the 2 studies suggest that the heritability of risk-taking behaviors in guppies may increase at adulthood, with this process occurring earlier in males than in females. Only a few studies have examined the contribution of additive variance to male and female personality traits separately, and those that did reported little difference in V_A_ of behavioral traits between the sexes. van Oers et al. (2004) reported similar amount of additive genetic variance in boldness and exploration for male and female great tits. In a captive guppy population, White et al. (2019) reported a similar genetic structure of risk-taking behavior in both sexes, although a G×S interaction was detected for 1 of the 4 behavioral metrics. In line with these reports, our study provides no clear evidence of a G × S interaction in boldness, although the trajectories of change in V_A_ indicate some potential for sex-specific patterns.

Maternal effects contributed substantially only to female boldness at time point 2 (7 to 8 months of age), which indicates that the effects of maternal investment may be expressed at specific life stages of daughters, including during adulthood. The result contrasts with the findings by White and Wilson (2019), who reported substantial maternal effects on the behavior of 1 to 2-month-old fish but not on adults. While the delayed expression of maternal effects found here may be surprising, their absence in older females (time points 3 and 4) is consistent with earlier studies on life-history traits. For example, Lindholm et al. (2006) observed a similar shift for body size in *Poecilia parae*, a species closely related to the guppy, while Wilson and Réale (2006) reported it for weight in sheep and cattle. This age-related decline is thought to reflect the diminishing importance of maternal effects as time passes since the mother's last influence on offspring. In contrast, maternal effects did not contribute substantially to male boldness at any life stage. Previous studies have reported sex-specific maternal effects on traits such as body size and developmental time in viviparous fish (Kruuk et al. 2015) and immune response in lizards (Svensson et al. 2009). However, evidence for sex-specific maternal effects on personality traits has been lacking until now. In our study, given the differing average boldness levels of males and females, it seems plausible that daughters benefit more from maternal influences on this behavioral trait than do sons, or that the effect in sons may be masked by other sources of variance. The evolutionary and proximate mechanisms underlying this sex-specificity, however, remain to be explored.

### Sex effect on boldness

Males in the experimental population were, on average, bolder than females, consistent with previous findings for Poeciliids (Brown et al. 2007), including guppies (Harris et al. 2010; Irving and Brown 2013; Kemp et al. 2022). Such sex-specific differences in trait levels may reflect sexually antagonistic selection arising from the contrasting life histories of the 2 sexes in this species (Magurran and Seghers 1994), resulting in different costs-benefit tradeoffs. Male guppies may benefit more from risky behavior because it enhances their mating opportunities, which they continually seek to maximize fitness. Guppy females have also been shown to prefer bold males (Godin and Dugatkin 1996) and bold males sire more offspring (Herdegen-Radwan 2019a). In line with this, while the ranking of individuals was maintained across contexts, males were generally less bold in the standard setup that in the other scenarios. Increased motivation to engage in risky behavior in the presence of a potential female partner may partly explain this modest change. In contrast, the benefits from repeated matings are expected to be lower for female guppies, which can store sperm and use it to produce subsequent broods. Instead, females are expected to invest more in survival—potentially requiring less risky behavior—to maximize their reproductive success, which increases with age as they grow larger. Thus, selection may act against boldness in females because of increased predation risk (Godin and McDonough 2003).

### Age-related changes in boldness

Average boldness declined significantly between time points 1 and 2, ie, between 4 to 5 and 7 to 8 months. As guppies mature at around 3 months of age, they were already sexually mature at the time of the first trial. However, developmental changes can continue after sexual maturity. Male guppies, which have determinate growth, experience most of their growth prior to maturation but continue to grow for up to 6 months after reaching sexual maturity, when they are considered fully mature adults. Therefore, the major change in boldness observed shortly after maturation might be related to the reorganization of physiological, metabolic, and hormonal systems around this crucial life stage. This pattern aligns with results from other studies on changes of behavioral types with age (reviewed by Cabrera et al. 2021), including fish, although these studies show mixed results in terms of the direction of the change. In Eastern mosquitofish, boldness was shown to increase around the time of maturation, but only in females (Vinogradov et al. 2022), while another study on the same species found no age effect on boldness (Polverino et al. 2016). Interestingly, a pattern of decreased boldness with age, similar to ours, was observed in a population of perch living under predation risk, whereas the opposite trend was found in their counterparts from a predation-free lake (Magnhagen and Borcherding 2008). As our population originated from a high-predation site, it is possible that the similar age-related decline observed in both studies reflects similar genetic mechanisms shaping boldness under predation pressure. Other behavioral traits along the proactive–reactive axis were also shown to shift between the juvenile and adult stages. While exploration and boldness both declined after maturity in killifish (Edenbrow and Croft 2011), as did activity in red squirrels (Kelley et al. 2015) and exploration in jounglefowl (Favati et al. 2016), the opposite was true for great tits who became more exploratory with age (Carere et al. 2005).

One possible explanation for the decline in boldness observed in our study is a decreased demand for resources at adulthood. Efficient resource acquisition allowing to meet energy needs of a growing organism are a priority mostly for young guppies, and bolder, risk-prone behavior correlates in the species with higher foraging efficiency (Fraser and Gilliam 1987). While in adulthood both males and females may still benefit from expressing risky behavior—either to seek partners or to forage—the early developmental stage, characterized by high investment in structural growth and energy storage, may be disproportionately demanding in terms of energy requirements (Martin et al. 2017).

We cannot entirely rule out another possibility, that the experimental design influenced both behavior and behavioral variation in the fish. Isolation is a significant stressor in social species, and in the present study, guppies were kept physically separated from conspecifics following the first boldness trial. If chronic stress induced behavioral changes, its largest effect on behavior might be expected between time points 1 and 2. However, stress is likely to be greatest immediately after isolation, ie between the first and second trials at time point 1. This would be expected to result in greatest decrease in boldness between those 2 trials and a possible disruption of repeatability at that stage, yet neither effect was observed. Importantly, the fish were kept in close proximity with visual contact with conspecifics, which should have mitigated the effects of isolation.

Interestingly, after a period of stability upon reaching adulthood, boldness increased again at older age, though it did not return to the original levels and the effect reached significance only in females. This pattern is in line with the terminal investment hypothesis (Hamilton 1966; Clutton-Brock 1984), which posits that older individuals, with limited prospects for survival, should invest more heavily in current reproduction. Empirical evidence for terminal investment has been found in several species (see meta-analysis by Foo et al. 2023). As boldness is positively correlated with male reproductive success in our population, an increase in boldness at older ages may support this increased investment. The reasons for terminal investment in females are less clear but may be related to benefits from increased foraging. As females grow throughout their lives, their energy demands are relatively large at old age, when they also invest most in reproduction, as bigger females produce larger broods. Females were also shown to exert control over brood retention (Evans et al. 2007) and new-born sizes (Barbosa and Magurran 2010), which could further explain variation in reproductive investment. While we did not allow for mating in this study, which limits direct testing of the terminal investment hypothesis, the heritability of boldness suggests that changes in boldness could still occur in the absence of immediate mating opportunities. In both sexes, mainly individual plasticity caused shifts in average boldness levels. However, as shown by the age-decomposition model, only in males were these shifts partly attributable to the selective disappearance of bolder individuals. Although a correlation between survival and boldness was detected in both sexes, higher male mortality likely generated sufficient variance for this effect to reach statistical significance. This selective disappearance of bolder males, occurring mostly at older ages, likely counteracted the plastic increase in boldness, making the overall change less pronounced in males than in females.

We found no evidence that genotype-by-age interactions contributed to changes in boldness at the individual level, as cross-age genetic correlations were all close to unity, indicating no rank changes of genotypes across life stages. G×A interactions are expected under evolutionary senescence scenarios, as a consequence of differential ageing among genotypes due to varying mutation loads. Such G×A interaction in personality traits has been reported in only a few studies, limited to 2 species of tits (Class and Brommer 2015, 2016; Class et al. 2019). In those cases, the behavioral traits declined with age in a genotype-dependent manner. The lack of evidence for behavioral senescence at the genetic level in our study underscores the importance of further investigations to evaluate whether the previously reported pattern is species-specific or not widely occurring across personality traits.

### Link between boldness and survival

We found evidence for a phenotypic-level correlation between boldness and survival, as indicated by the posterior distribution's central tendency being distinct from zero. While the 95% HPD interval for the posterior distribution of the correlation approached zero, and that for the regression of boldness on survival minimally overlapped it, both estimates of central tendency were clearly distinct from zero, supporting the presence of an effect. The direction of this relationship is consistent with a behavior-mediated life-history tradeoff, as bolder individuals had lower chances of surviving until the last behavioral trial, while earlier work has shown that bolder males have higher reproductive success (Herdegen-Radwan 2019a). These results support the conclusions of meta-analysis by Smith and Blumstein (2008), who reported positive effect of boldness on reproductive success but negative on survival (but see meta-analysis by Moiron et al. 2020). Our findings represent robust evidence for the presence of a behavior-linked life history tradeoff, as both correlations were found in the same population. Studies that looked at the effect of boldness on reproduction and survival in the same population are few and gave mixed results. In largemouth bass, Ballew et al. (2017) found similar pattern of boldness-mediated survival-reproduction tradeoff as our findings, while Réale et al. (2009) reported an opposite relationship, ie better survival of bolder bighorn sheep traded off with their reproductive success, but only at young age. Other studies found no support for boldness-mediated life history tradeoffs (Santicchia et al. 2018; Van de Walle et al. 2024). These mixed results suggest that the generality of behavior-mediated tradeoffs remains uncertain, highlighting the need for studies that examine the links between traits within the same populations.

Since there is no predation in our experimental population, the negative boldness-survival correlation cannot be explained by bolder individuals being more prone to predation, as seen in some wild populations (Ballew et al. 2017). Instead, this result indicates that laboratory populations may also harbor enough variation in resource allocation for the tradeoff to emerge. A potential explanation for the boldness-survival relationship is that bolder individuals may incur greater energy expenditures due to higher metabolic rates and oxidative stress related to higher reproductive effort, or may invest less in immune function, linking behavior and physiology as proposed by the POLS framework (Ricklefs and Wikelski 2002; Réale et al. 2010). Indeed, energy expenditure has been recognized as an important factor in ageing, influencing longevity across numerous species (Manini 2010). Since we did not measure metabolism in this study, the proximate mechanisms underlying this link remain to be explored. However, the explanation aligns with the negative association between body size and boldness found in males. The association suggests a tradeoff between investment in growth versus proactive behavior and a fast pace of life, as predicted by the POLS hypothesis, and is consistent with Pauli et al. (2019), who reported a similar pattern in size-selected medaka (Oryzias latipes) lines. The absence of the same effect in females is also consistent with a study on captive guppies, in which White et al. (2016) found no association between female risk-related behavior and growth rate. Finally, males in the present experiment survived shorter than females. As males on average also expressed higher boldness levels, the difference in lifespan may reflect their greater energetic investment in proactive behaviors, potentially leading to more rapid resource depletion.

While the experimental fish were kept in isolation and most of them were not allowed to reproduce (except the parents of F2), which limits the potential for competitive and reproductive effort in this cohort, the laboratory population from which they originated is maintained under conditions that allow competition for both food and mating partners, allowing for variation in energy expenditure. Given the genetic underpinning of both boldness and survival, as supported by their heritability in the studied population, even in the absence of immediate ecological triggers, differences in boldness and survival are thus expected to be expressed in the experimental fish and their association likely to be maintained. Such life-history-bahavior associations have been proposed by several hypotheses explaining maintenance of personality traits, including both genetic as well as purely phenotypic mechanisms. While the POLS framework is centered around the joint evolution of sets of traits (Ricklefs and Wikelski 2002; Réale et al. 2010), other scenarios—such as state-dependent life-history tradeoffs associated with behavioral type, as proposed in the asset-protection hypothesis (Wolf et al. 2007)—are also expected to support correlations among sets of traits at the phenotypic level.

Here, boldness and survival, both of which harbored nonzero additive genetic variance, also showed evidence of a genetic-level correlation, with central tendency estimates even higher than for the corresponding phenotypic correlation. POLS predictions rely on genetic link between the traits considered, since the hypothesis proposes the emergence of such correlations as an effect of joint evolution of the traits, either in form of correlational selection or negative pleiotropy. However, genetic correlations underlying POLS are often assumed based on phenotypic correlations but are rarely tested. Such assumption, ie that phenotypes are adequate predictors of genotypes, known as phenotypic gambit (Grafen 1984), may be justified in some, but not all cases. Most support for the association come from highly heritable traits (eg Chevarud 1988; Reusch and Blanckenhorn 1998; Réale and Festa-Bianchet 2000), while traits with low heritability and polygenic architecture may not follow the rule (Hadfield et al. 2007). Indeed, studies testing the phenotypic gambit in behavioral (Crespel et al. 2024) and ornamental (Hadfield et al. 2007) traits showed that phenotypic correlations are not always robust estimators of genetic correlations, and advocated caution in relying on phenotypic correlations. While our results suggest that the correlation between boldness and survival is present and consistent at both the phenotypic and genetic levels, it should be noted that such correspondence may be easier to capture in laboratory conditions, where environmental variance is reduced (Hadfield 2010). Nevertheless, in light of the previous study in this population, showing higher reproductive success of bolder guppies (Herdegen-Radwan 2019a), the present result supports the possibility of joint evolution of behavioral and life history traits, consistent with the POLS hypothesis, with boldness mediating the tradeoff between reproduction and survival. While the phenotypic correlation closely reflected the genetic correlation in our study, it remains important to examine genetic correlations directly when testing the POLS scenario, as the assumption of their joint evolution underlies the hypothesis.

## Conclusions

Long-term studies investigating performance across life stages can provide valuable insights into the complex interactions and changing contributions of factors that shape traits. Our study revealed highly dynamic, sex- and age-dependent patterns of boldness expression, which may be maintaining variation in behavioral traits at the population level. The link between boldness, reproductive success, and survival supports the role of personality in mediating life-history tradeoffs, providing another mechanism maintaining consistent differences in boldness across individuals, including within cohorts.

## Supplementary Material

arag020_Supplementary_Data

## Data Availability

Analyses reported in this article can be reproduced using the data provided by Herdegen-Radwan and Raubic (2026).
