# Supplementary material for: Age-related changes and selective disappearance shape variation in bold-shy continuum in guppies
Source: Behav Ecol. 2026 Feb 25;37(3):arag020. doi: 10.1093/beheco/arag020 (PMC13008831; doi:10.1093/beheco/arag020)
Supplement: arag020_Supplementary_Data [file arag020_supplementary_data.zip › Supplementary_Material_1.docx]

*Breeding design*

The breeding population is kept at sizes of several hundred individuals, which, combined with periodical intra-tank mixing, helps preserve substantial genetic variance (see e.g. Anonymous 2021). Males for the parental generation were taken at random from several tanks containing the stock population. Females were drawn from several same-age cohorts spanning approximately half a year of age and kept separated from other fish before maturity. The partners were expected to have a mean-population-level relationship to each other. The crosses of 50 males with 100 females, (two of them mated to each male) resulted in 283 offspring born to 53 mothers and 35 fathers, from which 257 (from 48 mothers) survived till behavioral trials. In the second round of matings, 17 F1 females gave birth to 166 F2 offspring sired by 11 F1 males, and 142 of those survived to the beginning of behavioral trials. The average brood size across the experiment was 6.7, with a minimum of 2 and a maximum of 15 offspring per brood. As a consequence of the mating design, full-sib as well as paternal half-sib families were present in both generations, while no maternal ones, as females gave birth to a single brood each. Across the pedigree, there were 59 full-sib families with 393 individuals having any full-sibs, and 17 paternal half-sib families. There were also 4 pairs of F2 families which produced cousins, a sister and a brother from F1 being mated each to an unrelated individual from a different F1 family. These matings resulted in 81 F2 individuals, each sharing both grandparents with their maternal or paternal cousins.
