# Supplementary material for: Age-related changes and selective disappearance shape variation in bold-shy continuum in guppies
Source: Behav Ecol. 2026 Feb 25;37(3):arag020. doi: 10.1093/beheco/arag020 (PMC13008831; doi:10.1093/beheco/arag020)

*Cross-context consistency of boldness*

*Methods*

Cross-context correlations were estimated to determine if and how individuals change their behavior across different contexts. To assess this, we employed a multivariate technique that combined behavioral data collected at time points 2 and 3 across three different contexts: standard, in the presence of a conspecific female, and with a novel object (see Methods). A character-state model (Model 1 in Tab.2) was fitted to estimate correlations in boldness across contexts, with the boldness scores repeatedly measured in the three contexts treated as a trivariate censored response variable. Boldness scores were right-censored at 300 s, corresponding to the maximum trial duration. Individuals that did not emerge from the shelter within the observation period were assigned a censoring time of 300 s and coded as right-censored. These data were analysed using a censored Gaussian distribution (family = "cengaussian"), which allows appropriate estimation of effects in the presence of censoring. Context-specific intercepts and fixed effects of age (time point), sex, and generation were included. To account for repeated measures, individual identity (ID) was included as a random effect with an unstructured variance–covariance matrix across contexts. Residual variances were allowed to differ between traits. Although allowing residual covariances across contexts would be biologically plausible, estimating these additional parameters reduced model robustness, leading to small effective sample sizes for some fixed effects. Importantly, estimates of both random and fixed effects were qualitatively unchanged under the simplified model. The strength of among-individual correlations between the response variables was estimated to evaluate the consistency of behavior across contexts.

*Results*

Among-individual correlations of boldness scores across contexts were high (Tab. S2.1, Fig. S2.1), indicating that the test captured a consistent behavioral trait across all three scenarios. Thus, we used results from all trials for the subsequent analyses and treated them as a single measure of boldness to increase the precision of our estimates. Full model is reported in Table S2.2.

Table S2.1 Among-individual correlation estimates of boldness across context pairs (Model 1 in Tab. 2). The ‘Standard’, ‘Female’, and ‘Object’ labels correspond to the respective setups of the emergence test. The table reports the median (and the mode) and 95% HPD interval of the posterior distributions.

| **Context pairs compared** | | **Estimate** | **95% HPDI** |
| --- | --- | --- | --- |
| *Standard - Female* |  | 0.941 (0.973) | [0.814 – 1.000] |
| *Female - Object* |  | 0.943 (0.975) | [0.823 – 1.000] |
| *Standard - Object* |  | 0.940 (0.973) | [0.809 – 1.000] |

Table S2.2 Results from Model 1 testing cross-context consistency of boldness. Boldness was modelled as a three-variate response, with levels corresponding to test contexts: ‘S’ (standard), ‘F’ (with female), and ‘O’ (with object). Variance components are reported in table A. Context-specific V_ID_ and COV_ID_ represent the variance and covariance of boldness at the among-individual (ID) level across contexts. Context-specific V_R_ represent the residual variances across contexts. Fixed effects of sex, age and generation on boldness in each context are reported in table B. Estimates are the means of the posterior distributions and are accompanied by 95% HPD intervals. Significant effects and variance components with posterior distributions non-overlapping zero are highlighted in bold. ESS denotes effective sample size.

|  | **Model term** | **Estimate ± HPDI** | **ESS** | ***pMCMC*** |
| --- | --- | --- | --- | --- |
| **A** | *V_ID_ boldness S* | **2409 [1391 – 3486]** | **>10 000** | **-** |
|  | *V_ID_ boldness F* | **3047 [1897 – 4249]** | **>10 000** | **-** |
|  | *V_ID_ boldness O* | **3174 [2010 – 4397]** | **>10 000** | **-** |
|  | *COV_ID_ boldness F-S* | **2482 [1711 – 3284]** | **>10 000** | **-** |
|  | *COV_ID_ boldness O-S* | **2528 [1752 – 3331]** | **>10 000** | **-** |
|  | *COV_ID_ boldness O-F* | **2862 [2040 – 3710]** | **>10 000** | **-** |
|  | *V_R_ boldness S* | **10684 [9414 – 11992]** | **>10 000** | **-** |
|  | *V_R_ boldness F* | **10213 [8951 – 11514]** | **>10 000** | **-** |
|  | *V_R_ boldness O* | **9889 [8647 – 11171]** | **>10 000** | **-** |
| **B** | *Boldness S* | **177.261 [161.64 – 193.59]** | **>10 000** | **<0.001** |
|  | *Boldness F* | **195.02 [178.84 – 211.24]** | **>10 000** | **<0.001** |
|  | *Boldness O* | **170.27 [154.18 – 186.41]** | **>10 000** | **<0.001** |
|  | *Boldness S x sex (M)* | **-42.74 [-60.90 - -24.60]** | **>10 000** | **<0.001** |
|  | *Boldness F x sex (M)* | **-48.54 [-67.19 - -30.04]** | **>10 000** | **<0.001** |
|  | *Boldness O x sex (M)* | **-35.61 [-54.09 - -17.04]** | **>10 000** | **<0.001** |
|  | *Boldness S x age (3)* | 8.04 [-7.13 – 23.41] | >10 000 | 0.301 |
|  | *Boldness F x age (3)* | -2.16 [-17.05 – 12.83] | >10 000 | 0.776 |
|  | *Boldness O x age (3)* | 3.00 [-11.70 – 17.69] | >10 000 | 0.689 |
|  | *Boldness S x generation (F2)* | 9.77 [-9.59 – 28.98] | >10 000 | 0.320 |
|  | *Boldness F x generation (F2)* | -1.87 [-21.57 – 17.85] | >10 000 | 0.852 |
|  | *Boldness O x generation (F2)* | 16.77 [-2.85 – 36.47] | >10 000 | 0.094 |

Figure S2.1. Posterior estimates (medians ± 95% HPDI) of among-individual correlations in boldness across combinations of emergence test contexts. Contexts are coded as ‘S’ (standard), ‘F’ (with female), and ‘O’ (with object).


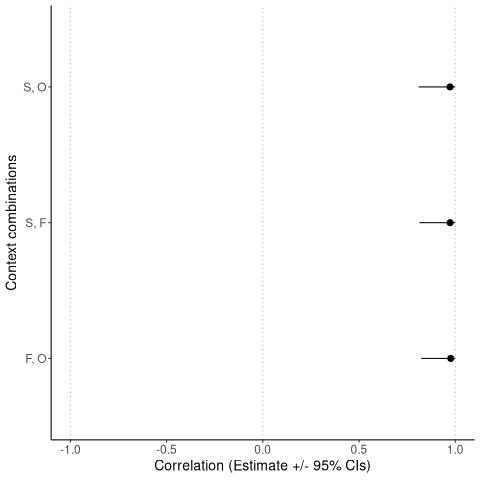

Supplement: arag020_Supplementary_Data [file arag020_supplementary_data.zip › Supplementary_Material_2.docx]
