# Supplementary material for: Age-related changes and selective disappearance shape variation in bold-shy continuum in guppies
Source: Behav Ecol. 2026 Feb 25;37(3):arag020. doi: 10.1093/beheco/arag020 (PMC13008831; doi:10.1093/beheco/arag020)
Supplement: arag020_Supplementary_Data [file arag020_supplementary_data.zip › Supplementary_Material_3.docx]

*Repeatability of boldness across time*

*Methods*

In sex-specific Models 2a (Tab. 2), all boldness scores at all four time points were used as response. Boldness scores were right-censored at 300 s, corresponding to the maximum trial duration. Individuals that did not emerge from the shelter within the observation period were assigned a censoring time of 300 s and coded as right-censored. These data were analysed using a censored Gaussian distribution (family = "cengaussian"), which allows appropriate estimation of effects in the presence of censoring. The model estimated individual-level variance-covariance matrix across time points. Residual (within-individual) variance was also modelled as age-specific, with residual covariances across time points constrained to zero. Age-specific repeatability was calculated as the proportion of among-individual variance relative to the phenotypic variance at a given time point (e.g. R=V_ID:AGE1_/V_P1_, for time point 1). The phenotypic variance (V_P_) was defined as the sum of the estimated variance components at that time point — i.e., among-individual and residual variance —conditional on the fixed effects. Fixed effects included age, generation, context and trial (the sequential number of repeated boldness tests within a time point).

Next, overall short- and long-term repeatability of boldness was calculated from a separate set of sex-specific models (Models 2b in Table 2). In these models, in addition to estimating the individual-level variance–covariance matrix for each time point (as described above), the overall cross-age effect of individual identity (V_ID_​) was estimated. This approach allowed us to partition variance due to age-specific effects from overall among-individual variance, and to calculate average repeatability across different time scales (Araya-Ajoy et al. 2015). Here, short-term repeatability was calculated as the sum of overall and age-specific among-individual variance (V_ID_+V_ID:AGE1-4_) across all time points, relative to the total phenotypic variance (V_P_). In other words, it represents the proportion of variance attributable to both overall and age-specific among-individual differences. Long-term repeatability was estimated as V_ID_/V_P_, i.e., the proportion of total phenotypic variance explained by among-individual variance. Unlike in Models 2a, in Models 2b phenotypic variance was calculated across all time points. Estimating short-term repeatability at each time point separately (Models 2a) allows us to assess how consistent individuals are at specific ages. In contrast, estimating both overall short-term repeatability and long-term repeatability (Models 2b) enables comparison of individual consistency across different time scales — within a single time point versus across the entire observation period.

*Results*

Short- and long-term repeatabilities are reported in Tab. S3.1. Full models are reported in Tab. S3.2 (Models 2a) and S3.3 (Models 2b).

Table S3.1 Estimates of repeatability (R) of boldness in males and females: A) short-term (*SH*) at each of the four ages (time points), derived from Models 2a (Tab. 2) ; B) short- and long-term (*SH* and *LH*, respectively) across time points, derived from Models 2b (Tab. 2). The table reports the median (±95% HPD interval) and the mode of the posterior distributions.

|  | **Timescale** |  | **R _females_** | **R _males_** |
| --- | --- | --- | --- | --- |
| **A** | *SH 1* |  | 0.244 [0.057 – 0.398]; 0.253 | 0.423 [0.210 – 0.572]; 0.435 |
|  | *SH 2* |  | 0.197 [0.102 – 0.298]; 0.193 | 0.276 [0.178 – 0.378]; 0.275 |
|  | *SH 3* |  | 0.235 [0.167 – 0.382]; 0.271 | 0.226 [0.131 – 0.327]; 0.223 |
|  | *SH 4* |  | 0.156 [0.044 – 0.281]; 0.148 | 0.348 [0.213 – 0.484]; 0349 |
| **B** | *SH* |  | 0.144 [0.090 – 0.207]; 0.139 | 0.154 [0.103 – 0.217]; 0.148 |
|  | *LN* |  | 0.032 [0.018 – 0.047]; 0.032 | 0.063 [0.044 – 0.083]; 0.062 |

Table S3.2 Results from Models 2a presented for females and males. Variance components are reported in table A. Time-specific V_ID_ and COV_ID_ represent among-individual variances and covariances of boldness across ages(time points), while time-specific V_R_ represent the residual (within-individual level) variances across time points. Fixed effects of age, generation, trial and context are reported in table B. The effect of context level ‘O’ could not be estimated due to high correlation with the reference level (‘F’). Estimates represent posterior means and are accompanied by 95% HPD intervals. Significant effects and variance components whose posterior distributions did not overlap zero are highlighted in bold. ESS denotes effective sample size.

|  | **Model term** | **Female estimate** | **ESS** | ***pMCMC*** | **Male estimate** | **ESS** | ***pMCMC*** |
| --- | --- | --- | --- | --- | --- | --- | --- |
| **A** | *V_ID_ age 1* | **3260 [598 – 5601]** | **>10 000** | **-** | **4175 [1808 – 6276]** | **>10 000** | **-** |
|  | *V_ID_ age 2* | **5375 [2454 – 8416]** | **>10 000** | **-** | **5548 [3196 – 8077]** | **>10 000** | **-** |
|  | *V_ID_ age 3* | **10552 [5722 – 15747]** | **>10 000** | **-** | **4997 [2550 – 7650]** | **>10 000** | **-** |
|  | *V_ID_ age 4* | **4242 [1014 – 7753]** | **>8 000** | **-** | **7768 [3909 – 12004]** | **>10 000** | **-** |
|  | *COV_ID_ age 1-2* | **2743 [1101 – 4430]** | **>10 000** | **-** | **2353 [951 – 3810]** | **>10 000** | **-** |
|  | *COV_ID_ age 1-3* | 1389 [-678 – 3489] | >10 000 | - | **2665 [1212 – 4170]** | **>10 000** | **-** |
|  | *COV_ID_ age 1-4* | -228 [-2056 – 1641] | >10 000 | **-** | **3493 [1740 – 5340]** | **>10 000** | **-** |
|  | *COV_ID_ age 2-3* | **6220 [3592 – 8985]** | **>10 000** | **-** | **5071 [3256 – 7013]** | **>10 000** | **-** |
|  | *COV_ID_ age 2-4* | **2927 [765 – 5187]** | **>10 000** | **-** | **6316 [3952 – 8865]** | **>10 000** | **-** |
|  | *COV_ID_ age 3-4* | **6023 [2906 – 9299]** | **>10 000** | **-** | **6048 [3644 – 8618]** | **>10 000** | **-** |
|  | *V_R_ age 1* | **10350 [7905 – 26980]** | **>10 000** | **-** | **5923 [4365 – 7868]** | **>10 000** | **-** |
|  | *V_R_ age 2* | **21531 [17718 – 25508]** | **>10 000** | **-** | **14386 [11937 – 16943]** | **>10 000** | **-** |
|  | *V_R_ age 3* | **27818 [22203 – 33725]** | **>10 000** | **-** | **16770 [13772 – 19893]** | **>10 000** | **-** |
|  | *V_R_ age 4* | **22126 [16863 – 27697]** | **>10 000** | **-** | **14325 [10679 – 18235]** | **>10 000** | **-** |
| **B** | *Intercept* | **73.17 [34.94 – 111.32]** | **>10 000** | **<0.001** | **47.37 [14.65 – 80.02]** | **>10 000** | **0.005** |
|  | *Age (2)* | **121.46 [97.29 – 145.82]** | **>10 000** | **<0.001** | **93.66 [71.83 – 115.50]** | **>10 000** | **<0.001** |
|  | *Age (3)* | **148.64 [119.21 – 178.37]** | **>10 000** | **<0.001** | **89.62 [66.94 – 112.41]** | **>10 000** | **<0.001** |
|  | *Age (4)* | **100.68 [76.11 – 125.16]** | **>10 000** | **<0.001** | **73.00 [80.59 – 95.55]** | **>10 000** | **<0.001** |
|  | *Generation (F2)* | -7.46 [-28.24 – 13.21] | >10 000 | 0.478 | -4.58 [-27.49 – 18.33] | >10 000 | 0.694 |
|  | *Trial (2)* | **47.23 [29.88 – 64.61]** | **>10 000** | **<0.001** | **30.10 [15.77 – 44.39]** | **>10 000** | **<0.001** |
|  | *Trial (3)* | 22.12 [-7.49 – 51.68] | >10 000 | 0.142 | 18.02 [-6.66 – 42.62] | >10 000 | 0.151 |
|  | *Context (S)* | 24.12 [-5.41 – 53.67] | >10 000 | 0.109 | **25.54 [0.88 – 50.30]** | **>10 000** | **0.042** |

Table S3.3 Results from Models 2a presented for females and males. Variance components are reported in table A. V_ID_ represents overall among-individual variance. Time-specific V_ID_ and COV_ID_ represent among-individual variances and covariances of boldness across ages (time points), while time-specific V_R_ represent the residual (within-individual level) variances across time points. Fixed effects of age, generation, trial and context are reported in table B. The effect of context level ‘O’ could not be estimated due to high correlation with the reference level (‘F’). Estimates represent posterior means and are accompanied by 95% HPD intervals. Significant effects and variance components whose posterior distributions did not overlap zero are highlighted in bold. ESS denotes effective sample size.

|  | **Model term** | **Female estimate** | **ESS** | ***pMCMC*** | **Male estimate** | **ESS** | ***pMCMC*** |
| --- | --- | --- | --- | --- | --- | --- | --- |
| **A** | *V_ID_* | **3071 [1809 – 4505]** | **>10 000** | **-** | **3851 [2735 – 5038]** | **>10 000** | **-** |
|  | *V_ID_ age 1* | **1260 [0.36 – 2687]** | **>10 000** | **-** | **1028 [0.46 – 2115]** | **>10 000** | **-** |
|  | *V_ID_ age 2* | **927 [0.20 – 3058]** | **>10 000** | **-** | **1960 [247 – 3891]** | **>10 000** | **-** |
|  | *V_ID_ age 3* | **5769 [1317 – 10726]** | **>10 000** | **-** | **1234 [0.40 – 2852]** | **>10 000** | **-** |
|  | *V_ID_ age 4* | **3170 [302 – 6434]** | **>10 000** | **-** | **1547 [0.30 – 3783]** | **>10 000** | **-** |
|  | *COV_ID_ age 1-2* | -610 [-1823 – 432] | >10 000 | - | **-1246 [-2097 - -449]** | **>10 000** | **-** |
|  | *COV_ID_ age 1-3* | **-2235 [-4077 - -533]** | **>10 000** | **-** | **-945 [-1770 - -187]** | **>10 000** | **-** |
|  | *COV_ID_ age 1-4* | **-1743 [-3348 - -248]** | **>10 000** | **-** | **-1031 [-1984 - -146]** | **>10 000** | **-** |
|  | *COV_ID_ age 2-3* | 1867 [-485 – 4604] | >9 800 | **-** | **1436 [134 – 2937]** | **>10 000** | **-** |
|  | *COV_ID_ age 2-4* | 1214 [-439 – 3136] | >10 000 | - | **1613 [42 – 3463]** | **>10 000** | **-** |
|  | *COV_ID_ age 3-4* | **3972 [1207 – 6988]** | **>10 000** | **-** | 1269 [-47 – 2926] | >10 000 | **-** |
|  | *V_R_ age 1* | **9591 [7621 – 11682]** | **>10 000** | **-** | **5594 [4423 – 6846]** | **>10 000** | **-** |
|  | *V_R_ age 2* | **22216 [18427 – 26179]** | **>10 000** | **-** | **14314 [11891 – 16867]** | **>10 000** | **-** |
|  | *V_R_ age 3* | **28001 [22371 – 33964]** | **>10 000** | **-** | **16780 [13783 – 19926]** | **>10 000** | **-** |
|  | *V_R_ age 4* | **22396 [16934 – 28181]** | **>10 000** | **-** | **14613 [10935 – 18542]** | **>10 000** | **-** |
| **B** | *Intercept* | **72.46 [34.31 – 110.41]** | **>10 000** | **<0.001** | **47.91 [15.46 – 80.47]** | **>10 000** | **0.004** |
|  | *Age (2)* | **120.83 [96.63 – 145.12]** | **>10 000** | **<0.001** | **93.49 [71.64 – 115.44]** | **>10 000** | **<0.001** |
|  | *Age (3)* | **147.06 [117.60 – 176.59]** | **>10 000** | **<0.001** | **89.45 [66.76 – 112.24]** | **>10 000** | **<0.001** |
|  | *Age (4)* | **101.58 [77.05 – 126.42]** | **>10 000** | **<0.001** | **72.80 [50.89 – 94.92]** | **>10 000** | **<0.001** |
|  | *Generation (F2)* | -4.48 [-25.55 – 16.64] | >10 000 | 0.675 | -3.74 [-26.55 – 19.11] | >10 000 | 0.747 |
|  | *Trial (2)* | **46.77 [29.88 – 63.67]** | **>10 000** | **<0.001** | **29.69 [15.78 – 43.71]** | **>10 000** | **<0.001** |
|  | *Trial (3)* | 21.88 [-7.68 – 51.28] | >10 000 | 0.145 | 17.49 [-6.96 – 41.88] | >10 000 | 0.160 |
|  | *Context (S)* | 24.02 [-5.41 – 53.45] | >10 000 | 0.109 | **25.04 [0.65 – 49.64]** | **>10 000** | **0.045** |

**Reference**

Araya-Ajoy, Y. G., K. J. Mathot, and N. J. Dingemanse. 2015. An approach to estimate short-term, long-term and reaction norm repeatability. Methods in Ecology and Evolution **6**:1462-1473.
