# Supplementary material for: Age-related changes and selective disappearance shape variation in bold-shy continuum in guppies
Source: Behav Ecol. 2026 Feb 25;37(3):arag020. doi: 10.1093/beheco/arag020 (PMC13008831; doi:10.1093/beheco/arag020)

Table S4.1 Results from Models 3a showing (A) variance components - additive genetic (V_A_), maternal identity (V_M_), individual identity (V_ID_) and residual (V_R_) - and (B) fixed effects of generation, age, context and trial on female and male boldness. COV terms represent the covariances in boldness at the respective levels across time points (age). The effect of context level ‘O’ could not be estimated due to high correlation with the reference level (‘F’). Estimates represent posterior means and are accompanied by 95% HPD intervals. Significant effects and variance components whose posterior distributions did not overlap zero are highlighted in bold. ESS denotes effective sample size.

|  | **Model term** | **Female estimate** | **ESS** | ***pMCMC*** | **Male estimate** | **ESS** | **pMCMC** |
| --- | --- | --- | --- | --- | --- | --- | --- |
| **A** | *V_ID_ age 1* | **2316 [0.29 – 4274]** | **>10 000** | **-** | **1116 [0.27 – 2606]** | **>10 000** | **-** |
|  | *V_ID_ age 2* | **341 [0.18 – 1357]** | **>10 000** | **-** | **1518 [0.23 – 3933]** | **>10 000** | **-** |
|  | *V_ID_ age 3* | **1245 [0.19 – 4138]** | **>10 000** | **-** | **1231 [0.21 – 3392]** | **>10 000** | **-** |
|  | *V_ID_ age 4* | **1188 [0.21 – 3570]** | **>10 000** | **-** | **1547 [0.23 – 4592]** | **>10 000** | **-** |
|  | *COV_ID_ age 1-2* | 108 [-1387 – 1681] | >10 000 | - | -888 [-2021 – 221] | >10 000 | - |
|  | *COV_ID_ age 1-3* | -1210 [-3219 – 701] | >10 000 | - | -778 [-1869 – 251] | >10 000 | - |
|  | *COV_ID_ age 1-4* | -1325 [-3161 – 382] | >10 000 | **-** | -820 [-2092 – 390] | >10 000 | - |
|  | *COV_ID_ age 2-3* | 128 [-1084 – 1586] | >10 000 | **-** | 1231 [-177 – 3323] | >9 000 | - |
|  | *COV_ID_ age 2-4* | 13 [-1242 – 1339] | >10 000 | **-** | 1403 [-205 – 3958] | >8 000 | - |
|  | *COV_ID_ age 3-4* | 961 [-549 – 3379] | >8 500 | - | 1255 [-206 – 3646] | >8 500 | - |
|  | *V_A_ age 1* | **968 [0.25 – 2474]** | **>10 000** | **-** | **3637 [1470 – 5899]** | **>10 000** | **-** |
|  | *V_A_ age 2* | **1782 [0.31 – 4110]** | **>10 000** | **-** | **3777 [666 – 6951]** | **>9 500** | **-** |
|  | *V_A_ age 3* | **9285 [3667 – 15397]** | **>10 000** | **-** | **4420 [1060 – 7865]** | **>10 000** | **-** |
|  | *V_A_ age 4* | **3622 [336 – 7289]** | **>10 000** | **-** | **6833 [1870 – 12181]** | **>10 000** | **-** |
|  | *COV_A_ age 1-2* | 1084 [-223 – 2658] | >9 000 | **-** | **3513 [1803 – 5349]** | **>10 000** | **-** |
|  | *COV_A_ age 1-3* | 2552 [-221 – 5245] | >10 000 | - | **3837 [2037 – 5784]** | **>10 000** | **-** |
|  | *COV_A_ age 1-4* | 1478 [-158 – 3167] | >10 000 | - | **4783 [2613 – 7155]** | **>10 000** | **-** |
|  | *COV_A_ age 2-3* | **3777 [477 – 7208]** | **>10 000** | **-** | **3993 [1061 – 6844]** | **>10 000** | **-** |
|  | *COV_A_ age 2-4* | **2277 [177 – 4515]** | **>10 000** | - | **4983 [1396 – 8631]** | **>10 000** | **-** |
|  | *COV_A_ age 3-4* | **5576 [1827 – 9539]** | **>10 000** | - | **5390 [1699 – 9087]** | **>10 000** | **-** |
|  | *V_M_ age 1* | **657 [0.21 – 1936]** | **>10 000** | **-** | **221 [0.18 – 856]** | **>10 000** | **-** |
|  | *V_M_ age 2* | **3871 [936 – 7186]** | **>10 000** | **-** | **408 [0.17 – 1555]** | **>10 000** | **-** |
|  | *V_M_ age 3* | **676 [0.19 – 2242]** | **>10 000** | **-** | **167 [0.17 – 712]** | **>10 000** | **-** |
|  | *V_M_ age 4* | **234 [0.18 – 873]** | **>10 000** | **-** | **218 [0.16 – 986]** | **>10 000** | **-** |
|  | *COV_M_ age 1-2* | 1136 [-471 – 2755] | >10 000 | **-** | 152 [-808 – 270] | >10 000 | - |
|  | *COV_M_ age 1-3* | 465 [-338 – 1651] | >10 000 | **-** | 35 [-390 – 519] | >10 000 | - |
|  | *COV_M_ age 1-4* | -26 [-843 – 735] | >10 000 | **-** | -40 [-585 – 394] | >10 000 | - |
|  | *COV_M_ age 2-3* | 1157 [-929 – 3503] | >10 000 | **-** | 10 [-597 – 650] | >10 000 | - |
|  | *COV_M_ age 2-4* | 113 [-1550 – 1920] | >10 000 | **-** | 167 [-394 – 1093] | >10 000 | - |
|  | *COV_M_ age 3-4* | 109 [-610 – 1008] | >10 000 | **-** | 79 [-309 – 637] | >10 000 | - |
|  | *V_R_ age 1* | **9858 [7675 – 12270]** | **>10 000** | **-** | **5777 [4512 – 7138]** | **>10 000** | **-** |
|  | *V_R_ age 2* | **20843 [17332 – 24511]** | **>10 000** | **-** | **14222 [11843 – 16719]** | **>10 000** | **-** |
|  | *V_R_ age 3* | **27424 [22061 – 33107]** | **>10 000** | **-** | **16628 [13632 – 19763]** | **>10 000** | **-** |
|  | *V_R_ age 4* | **21725 [16645 – 27097]** | **>10 000** | **-** | **14293 [10677 – 18199]** | **>10 000** | **-** |
| **B** | *Intercept* | **69.87 [30.80 – 108.79]** | **>10 000** | **<0.001** | **43.33 [8.13 – 78.37]** | **>10 000** | **0.016** |
|  | *Age (2)* | **118.49 [90.44 – 146.38]** | **>10 000** | **<0.001** | **92.33 [68.58 – 116.03]** | **>10 000** | **<0.001** |
|  | *Age (3)* | **144.12 [109.06 – 179.01]** | **>10 000** | **<0.001** | **89.31 [65.65 – 113.28]** | **>10 000** | **<0.001** |
|  | *Age (4)* | **97.53 [69.70 – 125.36]** | **>10 000** | **<0.001** | **72.39 [47.99 – 97.04]** | **>10 000** | **<0.001** |
|  | *Generation (F2)* | -0.52 [-27.88 – 26.49] | >10 000 | 0.974 | 11.21 [-14.52 – 36.95] | >10 000 | 0.390 |
|  | *Trial (2)* | **46.90 [29.89 – 63.92]** | **>10 000** | **<0.001** | **29.88 [15.82 – 44.09]** | **>10 000** | **<0.001** |
|  | *Trial (3)* | 21.94 [-7.22 – 51.11] | >10 000 | 0.140 | 17.56 [-6.82 – 42.08] | >10 000 | 0.159 |
|  | *Context (S)* | 24.03 [-5.07 – 53.18] | >10 000 | 0.106 | **25.48 [0.94 – 50.01]** | **>10 000** | **0.041** |

Table S4.2 Pairwise contrasts between age classes in predicted boldness, derived from Model 3a for females and males. The table reports the median of each contrast (±95% HPD interval) followed by *pMCMC* value corrected for multiple tests. Effects that remained significant after applying the Holmes-Bonferroni correction for multiple testing are highlighted in bold.

| **Time points** | **Females** | ***pMCMC*** | **Males** | ***pMCMC*** |
| --- | --- | --- | --- | --- |
| 1-2 | **-118.5 (-146.4 – -90.4)** | **>0.001** | **-92.3 (-116.0 – -68.6)** | **>0.001** |
| 1-3 | **-144.0 (-179.0 – -109.1)** | **>0.001** | **-89.3 (-113.3 – -65.5)** | **>0.001** |
| 1-4 | **-97.5 (-125.4 – -69.7)** | **>0.001** | **-72.3 (-97.0 – -48.0)** | **>0.001** |
| 2-3 | -25.6 (-56.9 – 5.5) | 0.207 | 3.0 (-16.2 – 22.2) | 0.753 |
| 2-4 | 21.0 (-13.2 – 55.0) | 0.226 | 20.0 (-6.7 – 46.6) | 0.429 |
| 3-4 | **46.6 (13.6 – 79.6)** | **0.018** | 17.0 (-10.1 – 43.9) | 0.437 |

Table S4.3 Results from Models 3b showing (A) variance components - individual identity (V_ID_) and residual (V_R_) - and (B) fixed effects of generation, age, trial, context and body size on female and male boldness. The effect of context level ‘O’ could not be estimated due to high correlation with the reference level (‘F’). Estimates represent posterior means and are accompanied by 95% HPD intervals. Significant effects and variance components whose posterior distributions did not overlap zero are highlighted in bold. ESS denotes effective sample size.

|  | **Model term** | **Female estimate** | **ESS** | ***pMCMC*** | **Male estimate** | **ESS** | ***pMCMC*** |
| --- | --- | --- | --- | --- | --- | --- | --- |
| **A** | *V_ID_* | **3777 [2512 – 5097]** | **>10 000** | ***-*** | **3942 [2780 – 5189]** | **>10 000** | ***-*** |
|  | *V_R_* | **21601 [19705 – 23612]** | **>8 000** | ***-*** | **13548 [12381 – 14796]** | **>10 000** | ***-*** |
| **B** | *Intercept* | **57.70 [13.48 – 103.89]** | **>10 000** | **0.015** | **36.27 [-2.15 – 74.63]** | **>10 000** | **0.064** |
|  | *Generation (F2)* | 0.90 [-32.08 – 33.58] | >10 000 | 0.964 | 2.42 [-21.75 – 26.13] | >10 000 | 0.832 |
|  | *Age (2)* | **121.14 [96.09 – 147.49]** | **>10 000** | **<0.001** | **92.37 [70.74 – 113.75]** | **>10 000** | **<0.001** |
|  | *Age (3)* | **136.53 [110.80 – 162.87]** | **>10 000** | **<0.001** | **87.32 [65.35 – 109.65]** | **>10 000** | **<0.001** |
|  | *Age (4)* | **94.15 [70.98 – 115.97]** | **>10 000** | **<0.001** | **67.63 [47.61 – 88.51]** | **>10 000** | **<0.001** |
|  | *Trial (2)* | **56.44 [34.28 – 78.94]** | **>10 000** | **<0.001** | **37.65 [18.21 – 56.83]** | **>10 000** | **<0.001** |
|  | *Trial (3)* | **32.43 [0.83 – 64.47]** | **>10 000** | **0.043** | **26.96 [0.55 – 54.23]** | **>10 000** | **0.048** |
|  | *Body size* | -3.71 [-19.05 – 12.25] | >10 000 | 0.642 | **14.65 [3.69 – 25.77]** | **>10 000** | **0.011** |
|  | *Context (S)* | **36.60 [4.82 – 68.22]** | **>10 000** | **0.021** | **33.38 [6.58 – 60.35]** | **>10 000** | **0.015** |

Table S4.4 Pairwise differences in the contribution of additive genetic variance to boldness across ages, estimated from Models 3a for females and males. The table reports posterior medians (±95% HPD interval) and modes. Differences with posterior distributions non-overlapping zero are highlighted in bold.

| **Time points** | **Females** | **Males** |
| --- | --- | --- |
| *1-2* | -690 [-3779 – 1882]; -339 | -89 [-4423 – 4093]; 25 |
| *1-3* | **-8171 [-14897 – -2110]; -7894** | -783 [-5152 – 3555]; -749 |
| *1-4* | -2540 [-7244 – 1656]; -2377 | -3196 [-19159 – 2677]; -2870 |
| *2-3* | **-7306 [-13551 –-1911]; -6936** | -632 [-3918 – 2579]; -567 |
| *2-4* | -1685 [-6398 – 2321]; -1270 | -2873 [-7449 – 903]; -2566 |
| *3-4* | 5534 [-280 – 11911]; 5242 | -2213 [-7052 – 1782]; -1901 |

Table S4.5 Sex differences in the contribution of additive genetic (GxS) and maternal identity (MxS) effects to boldness, estimated from sex-specific Models 3a. The table reports the posterior medians (±95% HPD intervals) and modes. Differences with posterior distributions non-overlapping zero are highlighted in bold.

| **Time point** | **GxS** | **MxS** | |
| --- | --- | --- | --- |
| *1* | 2669 [-47 – 5387]; 2647 | -298 [-2037 – 828]; -8 |  |
| *2* | 2037 [-2042 – 5972); 2122 | **-3340 [-6937** – **-200]; -8** |  |
| *3* | -4733 [-11806 – 1805]; -4486 | -283 [-2478 – 810]; -565 |  |
| *4* | 3185 [-3115 – 9571]; 3120 | -24 [-1144 – 1128]; -2 |  |

Table S4.3 Results from Models 3b showing (A) variance components - individual identity (V_ID_) and residual (V_R_) - and (B) fixed effects of generation, age, trial, context and body size on female and male boldness. The effect of context level ‘O’ for was not estimable due to high correlation with the reference level (‘F’). Estimates are posterior means accompanied by 95% HPD intervals. Significant effects are highlighted in bold. ESS denotes effective sample size.

|  | **Model term** | **Female estimate** | **ESS** | ***pMCMC*** | **Male estimate** | **ESS** | ***pMCMC*** |
| --- | --- | --- | --- | --- | --- | --- | --- |
| **A** | *V_ID_* | **3777 [2512 – 5097]** | **>10 000** | ***-*** | **3942 [2780 – 5189]** | **>10 000** | ***-*** |
|  | *V_R_* | **21601 [19705 – 23612]** | **>8 000** | ***-*** | **13548 [12381 – 14796]** | **>10 000** | ***-*** |
| **B** | *Intercept* | **57.70 [13.48 – 103.89]** | **>10 000** | **0.015** | **36.27 [-2.15 – 74.63]** | **>10 000** | **0.064** |
|  | *Generation (F2)* | 0.90 [-32.08 – 33.58] | >10 000 | 0.964 | 2.42 [-21.75 – 26.13] | >10 000 | 0.832 |
|  | *Age (2)* | **121.14 [96.09 – 147.49]** | **>10 000** | **<0.001** | **92.37 [70.74 – 113.75]** | **>10 000** | **<0.001** |
|  | *Age (3)* | **136.53 [110.80 – 162.87]** | **>10 000** | **<0.001** | **87.32 [65.35 – 109.65]** | **>10 000** | **<0.001** |
|  | *Age (4)* | **94.15 [70.98 – 115.97]** | **>10 000** | **<0.001** | **67.63 [47.61 – 88.51]** | **>10 000** | **<0.001** |
|  | *Trial (2)* | **56.44 [34.28 – 78.94]** | **>10 000** | **<0.001** | **37.65 [18.21 – 56.83]** | **>10 000** | **<0.001** |
|  | *Trial (3)* | **32.43 [0.83 – 64.47]** | **>10 000** | **0.043** | **26.96 [0.55 – 54.23]** | **>10 000** | **0.048** |
|  | *Body size* | -3.71 [-19.05 – 12.25] | >10 000 | 0.642 | **14.65 [3.69 – 25.77]** | **>10 000** | **0.011** |
|  | *Context (S)* | **36.60 [4.82 – 68.22]** | **>10 000** | **0.021** | **33.38 [6.58 – 60.35]** | **>10 000** | **0.015** |

Figure S4.1 Boldness data (raw scores), represented as scaled time to emerge from the shelter, shown in relation to age (time points 1 and 4) and trial. Males and females are plotted in separate panels. Boxes represent the **interquartile range (25th–75th percentiles),** the **line within each box indicates the median, whiskers extend to 1.5× the interquartile range**, and **dots represent outliers.** Significant differences between time points are indicated by asterisks (* p **< 0.05;** *** p **< 0.001**) above the horizontal bars.


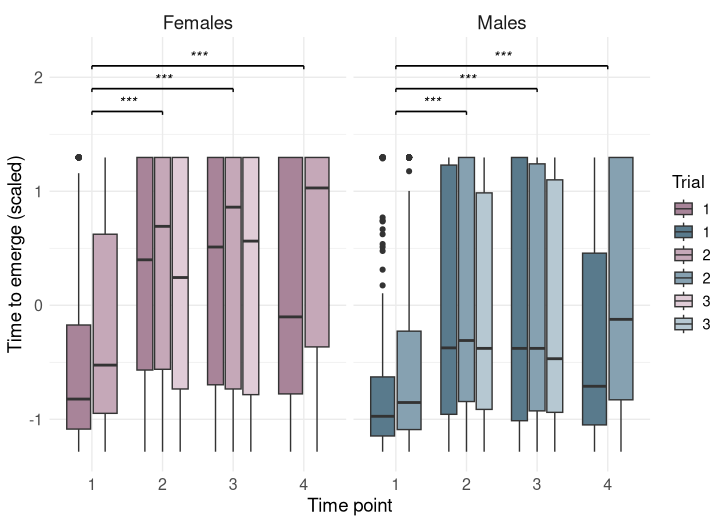

Supplement: arag020_Supplementary_Data [file arag020_supplementary_data.zip › Supplementary_Material_4.docx]
