# Supplementary material for: Age-related changes and selective disappearance shape variation in bold-shy continuum in guppies
Source: Behav Ecol. 2026 Feb 25;37(3):arag020. doi: 10.1093/beheco/arag020 (PMC13008831; doi:10.1093/beheco/arag020)
Supplement: arag020_Supplementary_Data [file arag020_supplementary_data.zip › Supplementary_Material_5.docx]

Table S5.1 Results from Model 4 testing phenotypic and genetic correlations between boldness (averaged scores from time point 1) and survival. V_A_ and V_R_ denote additive genetic and residual variance of the respective traits. COV_A_​ and COV_R_​ represent the additive genetic and residual covariances, respectively, between survival and boldness. Estimates represent posterior means accompanied by 95% HPD intervals. ESS denotes effective sample size. Significant effects and variance components with posterior distributions non-overlapping zero are highlighted in bold.

|  | **Model term** | **Estimate ± HPD interval** | **ESS** | ***pMCMC*** |
| --- | --- | --- | --- | --- |
|  | ***V_A_ survival*** | **2.50 [0.41 – 5.83]** | **>10 000** | **-** |
|  | ***V_A_ boldness*** | **0.38 [0.22 – 0.56]** | **>10 000** | **-** |
|  | *COV_A_ survival-boldness* | 0.37 [-0.03 – 0.86] | >10 000 | - |
|  | ***V_R_ survival*** | **3.33 [0.15 – 8.56]** | **>10 000** | **-** |
|  | ***V_R_ boldness*** | **0.66 [0.51 – 0.80]** | **>10 000** | **-** |
|  | *COV_R_ survival-boldness* | 0.03 [-0.40 – 0.45] | >10 000 | **-** |
|  | ***Survival*** | **3.27 [1.91 – 4.98]** | **>10 000** | **<0.001** |
|  | ***Boldness*** | **0.22 [0.01 – 0.43]** | **>10 000** | **0.041** |
|  | ***Survival x generation (F2)*** | **-2.00 [-3.30 – -0.82]** | **>10 000** | **<0.001** |
|  | *Boldness x generation (F2)* | -0.15 [-0.40 -0.11] | >10 000 | 0.254 |
|  | ***Survival x sex (M)*** | **-1.56 [-2.62 - -0.57]** | **>10 000** | **<0.001** |
|  | ***Boldness x sex (M)*** | **-0.39[-0.59 - -0.20]** | **>10 000** | **<0.001** |

*Age decomposition model*

Models 3c were similar to Models 3b, excluding the effect of size, but the effect of age was partitioned, for each individual, into a between-individual component (*Age M*), representing each individual's average age across observations, and a within-individual component (*Age WH*), representing deviations from the individual's mean age (Van de Pol and Wright, 2009). This approach distinguishes within-individual age-related change (*Age WH*​), indicative of phenotypic plasticity, from between-individual effects (*Age M*​), which reflect selective disappearance of some classes of individuals.

Table S5.2 Results from the within–between age decomposition model (Model 3c) for females (A) and males (B). V_ID_ and V_R_ denote individual and residual variance, respectively. The effect of context level ‘O’ could not be estimated due to high correlation with the reference level (‘F’). Estimates represent posterior means and are accompanied by 95% HPD intervals. Significant effects and variance components whose posterior distributions did not overlap zero are highlighted in bold. ESS denotes effective sample size.

|  | **Model term** | **Estimate ± 95% HPDI** | **ESS** | ***pMCMC*** |
| --- | --- | --- | --- | --- |
| **A** | ***V_ID_*** | **3700 [2461 - 85904]** | **>10 000** | **-** |
|  | ***V_R_*** | **22397 [20387 – 24421]** | **>10 000** | **-** |
|  | ***Intercept*** | **168.3 [79.0 – 258.1]** | **>10 000** | **<0.001** |
|  | ***Age WH*** | **31.3 [23.9 – 38.5]** | **>10 000** | **<0.001** |
|  | *Age M* | 24.7 [-10.3 – 60.2] | >10 000 | 0.171 |
|  | *Generation (F2)* | -4.6 [-27.5 – 18.4] | >10 000 | 0.695 |
|  | *Trial (2)* | 15.5 [-4.4 – 35.0] | >10 000 | 0.122 |
|  | *Trial (3)* | -8.1 [-38.3 – 22.5] | >10 000 | 0.560 |
|  | **Context S** | **-46.8 [-70.1 - -23.8]** | **>10 000** | **<0.001** |
| **B** | ***V_ID_*** | **3998 [2803 – 5256]** | **>10 000** | - |
|  | ***V_R_*** | **14122 [12845 – 83410]** | **>10 000** | - |
|  | ***Intercept*** | **86.6 [15.3 – 158.1]** | **>10 000** | **0.017** |
|  | ***Age WH*** | **20.7 [14.1 – 27.3]** | **>10 000** | **<0.001** |
|  | ***Age M*** | **28.9 [0.5 – 57.7]** | **>10 000** | **0.04** |
|  | *Generation (F2)* | **7.9 [-16.8 – 32.5]** | >10 000 | 0.528 |
|  | *Trial (2)* | 7.2 [-9.8 – 24.2] | >10 000 | 0.406 |
|  | *Trial (3)* | -4.5 [-30.7 – 20.5] | >10 000 | 0.728 |
|  | **Context S** | **-26.0 [-45.6 - -5.9]** | **>10 000** | **0.010** |

**Reference**

Van de Pol, M. and J. Wright. 2009. A simple method for distinguishing within- versus between-subject effects using mixed models. Animal Behaviour **77**:753-758.
